# Cyclosporine A Impairs Nucleotide Binding Oligomerization Domain (Nod1)-Mediated Innate Antibacterial Renal Defenses in Mice and Human Transplant Recipients

**DOI:** 10.1371/journal.ppat.1003152

**Published:** 2013-01-31

**Authors:** Emilie Tourneur, Sanae Ben Mkaddem, Cécilia Chassin, Marcelle Bens, Jean-Michel Goujon, Nicolas Charles, Christophe Pellefigues, Meryem Aloulou, Alexandre Hertig, Renato C. Monteiro, Stephen E. Girardin, Dana J. Philpott, Eric Rondeau, Carole Elbim, Catherine Werts, Alain Vandewalle

**Affiliations:** 1 INSERM U773, Centre de Recherche Biomédicale Bichat Beaujon, Université Paris 7 - Denis Diderot, Paris, France; 2 INSERM U699, Paris, France; Université Paris 7 - Denis Diderot, Paris, France; 3 Université de Poitiers, CHU Poitiers; Service d'Anatomie et Cytologie Pathologiques, Poitiers, France; 4 Service Urgences Néphrologiques et Transplantation Rénale and INSERM U702, Hôpital Tenon; Université Paris 6 - Pierre et Marie Curie, Paris, France; 5 Department of Laboratory Medicine and Pathobiology, University of Toronto, Toronto, Canada; 6 Department of Immunology, University of Toronto, Toronto, Canada; 7 INSERM UMR-S 945, Hôpital Pitié-Salpêtrière, Université Paris 6 - Pierre et Marie Curie, Paris, France; 8 Institut Pasteur, G5 Biologie et Génétique des Parois Bactériennes, Paris, France; Tufts University School of Medicine, United States of America

## Abstract

Acute pyelonephritis (APN), which is mainly caused by uropathogenic *Escherichia coli* (UPEC), is the most common bacterial complication in renal transplant recipients receiving immunosuppressive treatment. However, it remains unclear how immunosuppressive drugs, such as the calcineurin inhibitor cyclosporine A (CsA), decrease renal resistance to UPEC. Here, we investigated the effects of CsA in host defense against UPEC in an experimental model of APN. We show that CsA-treated mice exhibit impaired production of the chemoattractant chemokines CXCL2 and CXCL1, decreased intrarenal recruitment of neutrophils, and greater susceptibility to UPEC than vehicle-treated mice. Strikingly, renal expression of Toll-like receptor 4 (Tlr4) and nucleotide-binding oligomerization domain 1 (Nod1), neutrophil migration capacity, and phagocytic killing of *E. coli* were significantly reduced in CsA-treated mice. CsA inhibited lipopolysaccharide (LPS)-induced, Tlr4-mediated production of CXCL2 by epithelial collecting duct cells. In addition, CsA markedly inhibited Nod1 expression in neutrophils, macrophages, and renal dendritic cells. CsA, acting through inhibition of the nuclear factor of activated T-cells (NFATs), also markedly downregulated Nod1 in neutrophils and macrophages. Silencing the NFATc1 isoform mRNA, similar to CsA, downregulated Nod1 expression in macrophages, and administration of the 11R-VIVIT peptide inhibitor of NFATs to mice also reduced neutrophil bacterial phagocytosis and renal resistance to UPEC. Conversely, synthetic Nod1 stimulating agonists given to CsA-treated mice significantly increased renal resistance to UPEC. Renal transplant recipients receiving CsA exhibited similar decrease in NOD1 expression and neutrophil phagocytosis of *E. coli*. The findings suggest that such mechanism of NFATc1-dependent inhibition of Nod1-mediated innate immune response together with the decrease in Tlr4-mediated production of chemoattractant chemokines caused by CsA may contribute to sensitizing kidney grafts to APN.

## Author Summary

Patients who have received a kidney graft are treated with immunosuppressive drugs, such as the cyclosporine A (CsA). Transplanted patients under CsA are prone to bacterial infections. In this study, we used an experimental mouse model of kidney infection with *Escherichia coli* (*E. coli*) bacteria to study the effect of CsA. We show that CsA treatment of mice reduced their renal defense against *E. coli*. We found that CsA, in addition to its inhibitory action on the TLR4-mediated production of chemoattractant chemokines, also inhibited the expression of nucleotide-binding oligomerization domain 1 (Nod1), an intracellular receptor involved in the innate immune response against bacteria, in phagocytic cells. CsA acts by inhibiting the functions of the transcription factor NFAT. We show that NFAT is required for the proper expression of Nod1. Since Nod1 has already been reported to be involved in the phagocytic functions of polymorphonuclear neutrophils, we looked for and found a severe defect in neutrophil bacterial killing associated with reduced expression of Nod1 in both mice and patients treated by CsA. Interestingly, when mice treated with CsA are given synthetic molecules known to bind Nod1, this permitted the restoration of the Nod1 expression and renal defenses. This paper describes a novel mechanism which may explain, at least in part, why transplant patients receiving CsA have increased susceptibility to bacterial infection, and also provides a potential therapeutic strategy to restore renal antibacterial defenses.

## Introduction

Urinary tract infection (UTI) often complicated by acute pyelonephritis (APN), which is mainly caused by uropathogenic *Escherichia coli* (UPEC), is the most frequent infectious complication following renal transplantation [1], [2]. Despite improvement of the surgical procedures and the use of post-operative antibiotic prophylaxis, the rates of post-graft APN still remain higher than in the normal population [2], and late UTI occurring after more than 6 months after transplantation are associated with increased risk of death, and post-graft APN may also compromise long-term graft outcome [3], [4]. Although many factors including age, sex, and co-morbidity conditions play a role in the susceptibility to infection, long-term immunosuppressive therapy used to prevent episodes of acute graft rejection obviously increases the risk of bacterial, viral or fungal infections in the context of transplantation [5], [6].

Calcineurin inhibitors, such as Cyclosporine A (CsA), are almost incontrovertible drugs widely used to prevent renal graft rejection. Their main function is to inhibit the phosphatase activity of calcineurin, which regulates the nuclear translocation of the nuclear factor of activated T-cells (NFATs) transcription factor [7]. Impaired activation of NFATs then prevents the transcription of cytokine genes, including IL-2, in activated T cells [8]. However, the mechanism(s) by which CsA could alter the innate immune system, and thereby decrease renal host defenses against ascending UPEC remain largely unknown.

Early recognition of bacterial motifs by a number of pattern recognition receptors, including Toll-like receptors (TLRs) and (Nod)-like receptors (NLRs), is essential for the removal of bacterial pathogens [9]. UPEC colonizing the urinary tract are recognized by several TLRs, including TLR2, 4, 5, and 11 [10]. Studies using experimental murine models of ascending UTI have demonstrated that Tlr4, which senses lipopolysaccharide (LPS) from Gram-negative bacteria [10], and also Tlr11, that is expressed in murine bladder epithelial cells and RTECs [11], regulate susceptibility to UTIs in mice. TLRs play key roles in activating the transcription factor NF-κB and the mitogen-activated protein kinases (MAPKs) signaling pathways leading to the production of chemoattractant cytokines and subsequent recruitment of neutrophils and monocytes/macrophages for efficient clearance of the bacteria. Nod1 and Nod2 also promote the activation of NF-κB and MAPKs through the recruitment of the kinase RIP-2 (also known as RIP2K or RICK), which is a member of the caspase activation and recruitment domain (CARD) protein family [12], [13]. Nod1 recognizes muramyl tripeptide (M-TriDAP), a degradation product of peptidoglycan (PGN) containing DAP which is present in most Gram-negative bacteria and some Gram-positive bacteria [14], [15], while Nod2 recognizes muramyl dipeptide (MDP), a motif common to PGNs from all classes of bacteria [15]. Nod2 is mainly expressed in monocytes and macrophages, and mutations of *NOD2* are associated with Crohn's disease, an inflammatory bowel disease mainly driven by T cells [16], [17]. The functions of Nod1, which is more ubiquitously expressed, differ somewhat from those of Nod2. Recent studies have demonstrated that Nod1 plays a key role in the migration of neutrophils into the intestine and liver [18], and in activating phagocytic mechanisms of bacterial killing [19], [20]. The fact that altered leukocyte functions and decreased capacity for bacterial phagocytosis are the most common abnormalities in the immune status of renal transplant recipients [21], [22], led us to investigate the possibility that CsA alters the Nod1-mediated neutrophil functions and bacterial phagocytic killing of UPEC.

In the present study, we used an experimental mouse model of ascending UTI and show that the administration of CsA to wild-type (WT) mice decreases renal resistance to UPEC infection. CsA impaired Tlr4-mediated activation and subsequent production of chemoattractant chemokines in the epithelial collecting duct cells, to which UPEC bind preferentially during their retrograde ascent along the urinary tract system [23]. In addition, CsA, through its inhibitory action on NFATs, also markedly inhibited the functional expression of Nod1 in phagocytic cells, including neutrophil migration capacity and phagocytic killing of UPEC. Similar to CsA-treated WT mice, *Nod1^−/−^* mice exhibited greater susceptibility to UPEC than their WT counterparts. Using 11R-VIVIT, a synthetic peptide inhibitor of NFATs [24], we also demonstrate *in vitro* and *in vivo* the relevance of the regulatory role of the NFATc1 isoform in controlling the Nod1-mediated renal susceptibility to UPEC. We also report the functional downexpression of human NOD1 and decreased phagocytic capacity of *E. coli* in leukocytes from renal transplant recipients treated with CsA. The combined inhibitory effects of CsA on Tlr4-mediated chemokine production and Nod1-mediated migration of neutrophils and bacterial phagocytic capacities, which contribute to decrease renal antibacterial defenses in mice, may explain, at least in part, the susceptibility of CsA-treated renal transplant recipients to bacterial infections.

## Results

### CsA increases susceptibility to renal retrograde infection by UPEC

**Figure 1:**
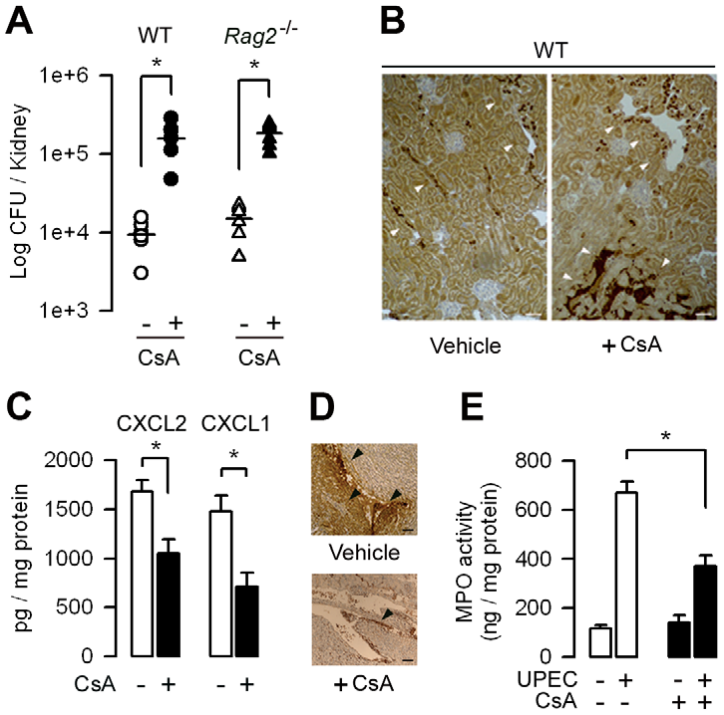
CsA decreases renal host resistance to UPEC. (A) Bacterial loads in kidneys from vehicle- (-) and CsA-treated (+) WT mice, and vehicle or CsA-treated *Rag2^−/−^* mice (*n* = 6–8 per group) 24 h after the transurethral inoculation of the UPEC strain HT7. Horizontal bars are the mean value of each group. (B) Illustrations of the intrarenal *E. coli* immunostaining in the infected kidneys from vehicle- and CsA-treated mice. Bars, 100 µm. (C) Production of CXCL2 and CXCL1 in the 24 h post-infected kidneys from vehicle-treated (−) and CsA-treated (+) mice. (D) Illustrations of neutrophils infiltrates (arrowheads) in the papilla from post-infected vehicle- and CsA-treated mice kidneys. Bars, 100 µm. (E) MPO activity in the 24 h post-infected kidney homogenates from vehicle- and CsA-treated mice. Values are presented as mean ± SE from 6–8 determinations per group. *, *p*<0.05 (Two-tailed, unpaired Student's *t* test).

WT mice treated with CsA (15 mg/kg) or its vehicle for 5 days were then infected by transurethral inoculation with the UPEC HT7 strain isolated from the urine of a woman with acute pyelonephritis [4], [25], to test whether CsA affected renal antibacterial defenses. 24 h after the inoculation of live UPEC, the bacterial burden and *E. coli* positive immunostaining were greater in CsA-treated mice than vehicle-treated mice (Figure 1A and B). As a control, we checked that CsA did not modify the growth rate of UPEC (not shown), thus excluding any direct effect of the calcineurin inhibitor on the bacteria. CsA also increased renal bacterial loads in kidneys from *Rag2^−/−^* mice, which lack mature lymphocytes, to almost the same extent as in WT kidneys (Figure 1A), suggesting that CsA promotes UPEC infection independently of its inhibitory effect on the adaptive immune system. The amount of secreted chemokines MIP-2/CXCL2 and KC/CXCL1, which play key roles in the chemoattraction of neutrophils during experimental UTI [23], [26]–[28], was also significantly lower in kidneys from CsA-treated mice than vehicle-treated mice (Figure 1C). Fewer Ly-6G^+^ neutrophils were detected in the kidney medulla and in the urinary space in the CsA-treated mice than in untreated mice (Figure 1D). Quantification of neutrophils assessed by measuring myeloperoxidase (MPO) activity also revealed significant lower MPO activity in the 24 h post-infected kidney homogenates from CsA-treated mice than vehicle-treated mice (Figure 1E).

### CsA impairs the migration of neutrophils and phagocytic killing of UPEC

**Figure 2:**
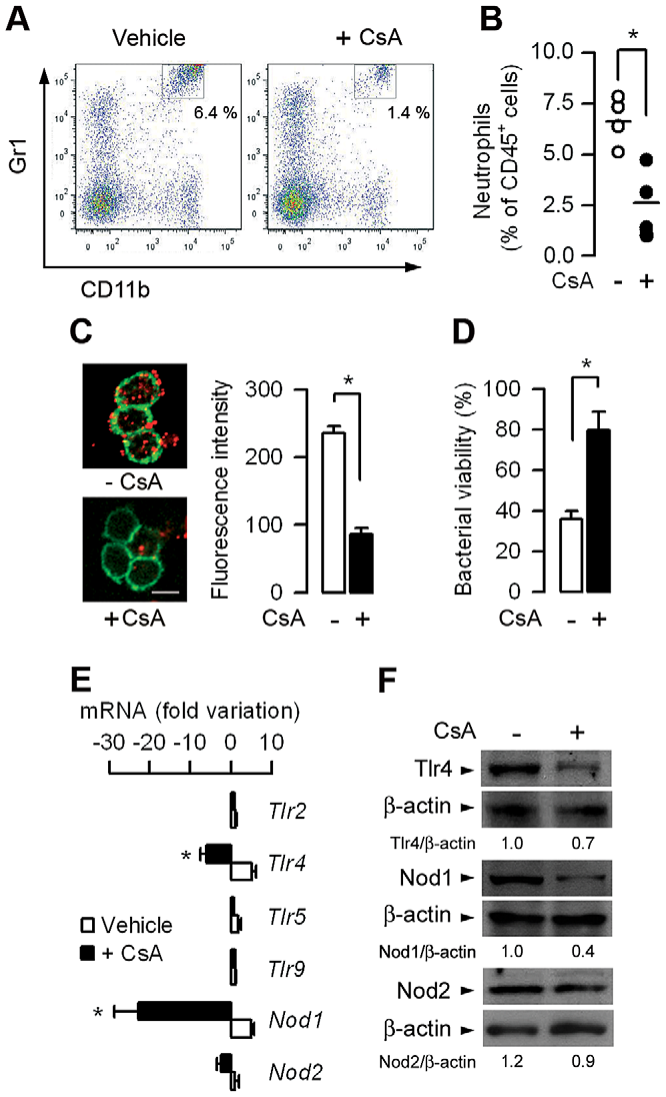
CsA impairs the migration of neutrophils and macrophages in UPEC-infected kidneys and neutrophil bacterial phagocytic killing capacity. (A and B) Flow cytometry analysis of the CD45^+^ leukocyte population infiltrating kidneys from CsA- and vehicle-treated mice, 24 h after UPEC inoculation. Representative dot-plots of CD45^+^ F4/80^−^ renal cells (A) and percentages (A, upper right insets and B) of F4/80^−^ CD11b^+^ Gr1^HI^ neutrophils infiltrating the infected CsA- and vehicle-treated mice kidneys. The bars indicate the mean percentage of neutrophils over total CD45^+^ leukocytes (*n* = 4 mice per group). (C) Illustrations and quantification of Texas red-coupled *E. coli* internalized by peritoneal neutrophils from vehicle-treated (−) and CsA-treated mice. Values are represented as mean ± SE of the mean count values (3–5 per condition) from three independent experiments. Cell membranes were stained with CD11b-FITC. Bars, 10 µm. (D) Killing of serum-opsonized *E. coli* by neutrophils from untreated or CsA-treated WT mice. Bacterial viability (*n* = 5–7 determinations from three experiments) was expressed relative to a control assay performed without neutrophils. (E) Variation in the levels of *Tlr*2, *Tlr4*, *Tlr5*, *Tlr9*, *Nod1*, and *Nod2* mRNAs measured by quantitative real time PCR in kidneys from CsA-treated (+) and vehicle-treated mice 24 h after UPEC inoculation. (*n* = 6–8 determinations from 3–4 experiments). (F) Immunoblot analysis of the Tlr4, Nod1, and Nod2 proteins and the corresponding ß-actin in kidney homogenates from vehicle-treated and CsA-treated mice 24 h after the inoculation of UPEC. Values are presented as mean ± SE. *, *p*<0.05 (B–D, Two-tailed, unpaired Student's *t* test; E, Mann-Whitney test).

Flow cytometry (FACS) analysis revealed that the CD45^+^ leukocyte population detected in the 24 h post-infected kidneys was essentially composed of F4/80^+^ CD11b^LO^ Gr1^−/LO^ MHC-II^+^ CD11c^+^ renal dendritic cells (DCs), which have been shown to form a contiguous network in the renal tubulointerstitium [29], F4/80^−^ CD11b^+^ Gr1^HI^ MHC-II^−^ CD11c^−^ neutrophils, and to a lesser extent F4/80^+^ CD11b^+^ Gr1^INT^ MHC-II^−^ CD11c^−^ inflammatory monocytes/macrophages. FACS analysis revealed a significant decrease in the proportion of neutrophils over the total CD45^+^ renal cell population detected in the 24 h post-infected kidneys from CsA-treated mice compared to vehicle-treated mice kidneys (Figure S1 and Figure 2A and B). In contrast, CsA only slightly, and non-significantly reduced the number of monocytes/macrophages or DCs present in the 24 h post-infected kidneys (Figure S1). This suggests that CsA preferentially impairs the migration capacity of neutrophils in the UPEC-infected kidneys. *In vitro* experiments using the Boyden chamber method also revealed that the migration capacity of neutrophils isolated from the blood of the 5-day CsA-treated mice and stimulated by the neutrophil activating agent N-formyl-methionyl leucyl-phenylalanine (fMLP) or by CXCL2 was significantly decreased compared to that of neutrophils from vehicle-treated WT mice (Figure S2A and B). CsA also altered the bacterial phagocytic killing capacity by neutrophils. In contrast to neutrophil-enriched peritoneal cells (NPCs) isolated from vehicle-treated mice, NPCs collected from CsA-treated mice exhibited significantly lower *ex vivo* capacity to internalize Texas red-coupled *E. coli* and kill serum-opsonized *E. coli* (Figure 2C and D).

*Nod1^−/−^* neutrophils were shown to exhibit deficient capacity of bacterial phagocytic killing and lower migration capacity than WT neutrophils [20], [30]. Given that neutrophil migration and their phagocytic killing capacities were markedly reduced in CsA-treated mice, we tested whether CsA directly alters intrarenal expression of Nod1. Quantitative real-time PCR revealed that the levels of *Nod1* mRNA expression and, to a lesser extent those of *Tlr4*, but not of *Nod2*, were markedly decreased in the 24 h post-infected CsA-treated mice kidneys compared to those of the infected vehicle-treated mice (Figure 2E). In accordance with these findings, the amount of the immunodetected Nod2 protein remained equivalent in the infected kidneys from CsA-and vehicle-treated mice, whereas the amounts of Nod1 and Tlr4 proteins were ∼50% and ∼30%, respectively, lower in the infected kidneys from CsA-treated mice than in those from their vehicle-treated counterparts (Figure 2F). Despite the decrease in Nod1 expression, the level of phosphorylated over total RIP-2, which is involved in the control of Nod-mediated NF-κB activation [12], was similar in the 24 h post-infected kidneys from CsA-treated mice and vehicle-treated mice (not shown). Collectively, these findings suggest that, in addition to an inhibitory effect on Tlr4 mRNA and protein expression, CsA impairs the recruitment and functions of neutrophils in the inflamed kidneys by a mechanism possibly linked to downregulation of Nod1 expression.

### Nod1 is involved in host renal defenses against UPEC

**Figure 3:**
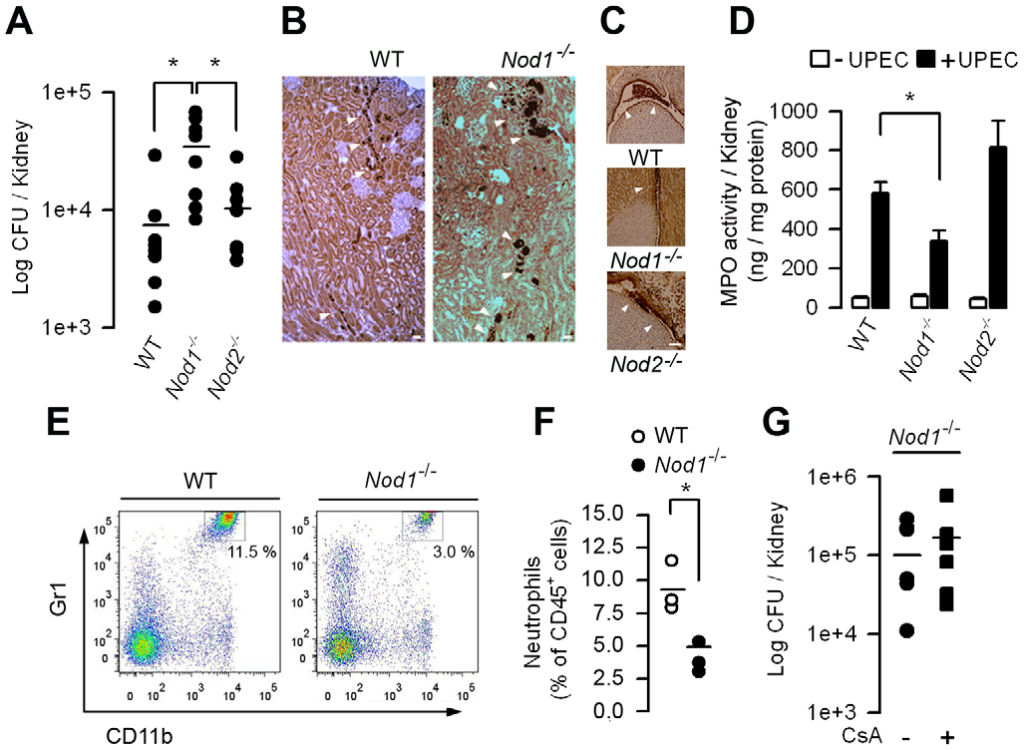
*Nod1^−/−^* mice display increased susceptibility to renal retrograde UPEC infection. (A) Bacterial loads in kidneys from WT, *Nod1^−/−^* and *Nod2^−/−^* mice (n = 8 per group) 24 h after the transurethral inoculation of UPEC strain HT7. Horizontal bars are the mean value of each group. (B) Illustrations of the *E. coli* immunostaining (arrowheads) in the infected WT and *Nod1^−/−^* mice kidneys. Bars = 100 µm. (C) Illustrations of neutrophils infiltrates (arrowheads) in the urinary space from post-infected WT, *Nod1^−/−^*, and *Nod2^−/−^* kidneys. Bars = 100 µm. (D) Levels of MPO activity in kidney homogenates from post-infected WT, *Nod1^−/−^*, and *Nod2^−/−^* mice. (E and F) Flow cytometry analysis of the CD45^+^ leukocyte population infiltrating the infected kidneys from WT and *Nod1^−/−^* mice 24 h after UPEC inoculation. Representative dot-plots of CD45^+^ F4/80^−^ renal cells (E) and percentages (E, upper right insets and F) of F4/80^−^ CD11b^+^ Gr1^HI^ neutrophils infiltrating the infected WT and *Nod1^−/−^* mice kidneys. The bars indicate the mean percentage of neutrophils over total CD45^+^ leukocytes (*n* = 3 mice per group. (G) Bacterial loads in kidneys from untreated (–) and day-5 *Nod1^−/−^* mice (n = 8 per group) 24 h after the transurethral inoculation of the UPEC strain HT7. Horizontal bars are the mean value of each group. Values are presented as mean ± SE. *, *p*<0.05 (A, D, Mann-Whitney test; F, G, Two-tailed, unpaired Student's *t* test).

We next investigated the consequence of Nod1 deficiency in host renal bacterial defenses. The renal bacterial burden and the number of immunodetected UPEC were significantly greater in the kidneys of *Nod1* ^−/−^ mice than in those of WT, 24 h after the inoculation of UPEC (Figure 3A and B). Less Ly-6G^+^ neutrophils were detected in the urinary space from post-infected *Nod1* ^−/−^ mice than from post-infected WT or *Nod2* ^−/−^ mice (Figure 3C), and, like in UPEC-infected CsA-treated mice, the MPO activity also remained significantly lower in the post-infected *Nod1^−/−^* than in post-infected WT or *Nod2^−/−^* kidneys (Figure 3D). FACS also showed that the proportion of polymorphonuclear neutrophils infiltrating the 24 h post-infected *Nod1* ^−/−^ mice kidneys was lower than in the WT kidneys (Figure 3E and F). In accordance with the findings of Dharancy *et al.* [30], *in vitro* experiments using a Boyden chamber revealed that the migration capacity of neutrophils isolated from naive *Nod1^−/−^* mice and activated by fMLP (or with CXCL2, not shown) was significantly lower than that of WT neutrophils (Figure S2C and D). These findings also suggested that Nod1 is implicated in the migration capacity of neutrophils. Consistently with a role for Nod1 in the bacterial phagocytic killing capacity of neutrophils [20] NPCs collected from *Nod1^−/−^* mice, but not those isolated from WT or *Nod2^−/−^* mice, were unable to internalize Texas red-coupled *E. coli* (Figure S3A and B). A significant reduction in the killing of serum-opsonized UPEC was also observed *in Nod1^−/−^* neutrophils compared to WT or *Nod2^−/−^* neutrophils (Figure S3C). We then analyzed the effects of CsA on the renal susceptibility of *Nod1^−/−^* mice to UPEC. Administration of CsA slightly, but not significantly, increased the renal bacterial loads in kidneys from *Nod1^−/−^* mice compared to those from untreated *Nod1^−/−^* mice (Figure 3G), further suggesting that CsA impairs Nod1-mediated antibacterial defenses to UPEC.

### CsA differently alters TLR4- and Nod-mediated signaling in renal tubule cells and neutrophils, macrophages, or renal dendritic cells activated by UPEC

**Figure 4:**
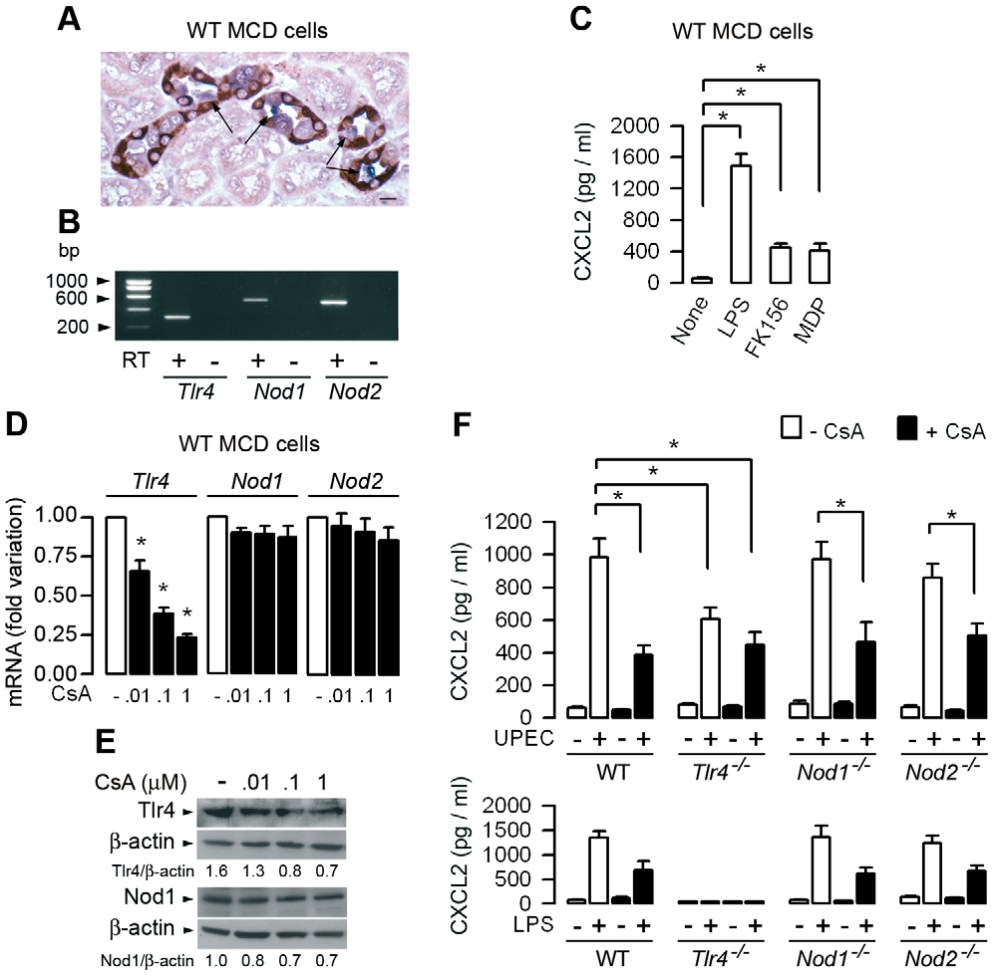
CsA impairs Tlr4-mediated chemokine production in renal medullary collecting duct cells. (A) Illustration showing UPEC (in blue) adhering to the luminal surface of cells from collecting duct sections (arrows), identified by the AQP-2 positive staining of the collecting duct principal cells (in dark red). Bar = 20 µm. (B) Identification by reverse transcription PCR of amplified products of *Tlr4* (311 bp), *Nod1* (525 bp), and *Nod2* (507 bp) mRNAs in a confluent culture of medullary collecting ducts (MCDs) dissected from the kidney of a WT mouse. (C) CXCL2 production in cultured WT MCD cells incubated with or without LPS, FK156, or MDP for 6 h at 37°C. (D) Relative levels of *Tlr4*, *Nod1* and *Nod2* mRNAs measured by quantitative real time PCR in confluent cultures of WT MCD cells incubated with various concentrations of CsA for 48 h and compared to untreated MCD cells. (E) Immunoblot analysis of Tlr4 and Nod1, and the corresponding β-actin in WT MCD cells incubated with or without various concentrations of CsA for 48 h. (F) CXCL2 production in cultured MCD cells dissected from WT, *Tlr4^−/−^*, *Nod1^−/−^*, or *Nod2^−/−^* kidneys incubated with or without CsA for 48 h, then with or without 10^4^ UPEC (upper panel, n = 3–5 determinations from 3 independent experiments) or 10 ng/ml LPS (lower panel, values from 4 separate cultures of MCD cells dissected from a single mouse in each group) for 3 h or 6 h, respectively. Values are presented as mean ± SE. *, *p*<0.05 (C, E, Mann-Whitney test; D, Two-tailed, unpaired Student's *t* test).

Previous studies had demonstrated that bladder epithelial cells and renal epithelial tubule cells are actively involved, together with bone marrow-derived cells, in the chemoattraction of neutrophils to the site of inflammation in experimental models of ascending UTI [31], [32]. We also showed that UPEC preferentially binds to the apical side of epithelial cells constituting the terminal collecting duct (Figure 4A) [33]. Activation of TLR4 signaling in the urinary tract system infected by UPEC plays a key role in this process [23], [34]. Because renal tubule cells also express the Nod1 and Nod2 receptors, that can be activated in inflamed kidneys [35], [36], experiments were carried out to analyze the effects of CsA on both Tlr4, and Nod1 or Nod2 signaling in renal tubule cells and bone marrow-derived cells activated by LPS, Nod ligands or UPEC. We checked that the renal medullary collecting duct (MCD) cells did express *Tlr4*, *Nod1*, and *Nod2* mRNAs (Figure 4B). LPS, and to a much lesser extent the Nod1 agonist FK156 and the Nod2 agonist MDP, stimulate the production of CXCL2 in cultured WT MCD cells (Figure 4C). CsA inhibited in a dose-dependent manner the Tlr4 mRNA and protein expressions without altering Nod1 and Nod2 expressions (Figure 4D and E). We then tested whether 100 nM (corresponding to 120 ng/ml) CsA alters the TLR4- and/or Nod-mediated cellular response in MCD cells. The CXCL2 production caused by UPEC significantly decreased in untreated *Tlr4^−/−^* MCD cells compared to WT, *Nod1^−/−^*, or *Nod2^−/−^* MCD cells, and also decreased to almost the same extent in CsA-treated WT, *Nod1^−/−^*, or *Nod2^−/−^* MCD cells than in CsA-treated *Tlr4^−/−^* MCD cells (Figure 4F, upper panel). As controls, similar profiles of CXCL2 production were obtained by incubating WT, *Nod1^−/−^*, and *Nod2^−/−^* MCD cells with LPS, and no significant production of CXCL2 was detected in *Tlr4^−/−^* MCD cells (Figure 4F, lower panel). These findings thus suggest that CsA mainly affects the predominant TLR4-mediated production of CXCL2 and has only a minor effect on epithelial Nod1- and Nod2-mediated renal tubule cell activation caused by UPEC. CsA also significantly reduced the ability of LPS-activated confluent WT MCD cells, which developed high electrical transepithelial resistance (∼4500 Ω. cm^2^), to stimulate the *in vitro* migration capacity of neutrophils as compared to untreated WT MCD cells incubated with LPS (Figure S4A to C). As a control, *Tlr4* ^−/−^ MCD cells challenged with LPS did not stimulate the migration of neutrophils (Figure S4B and C).

**Figure 5:**
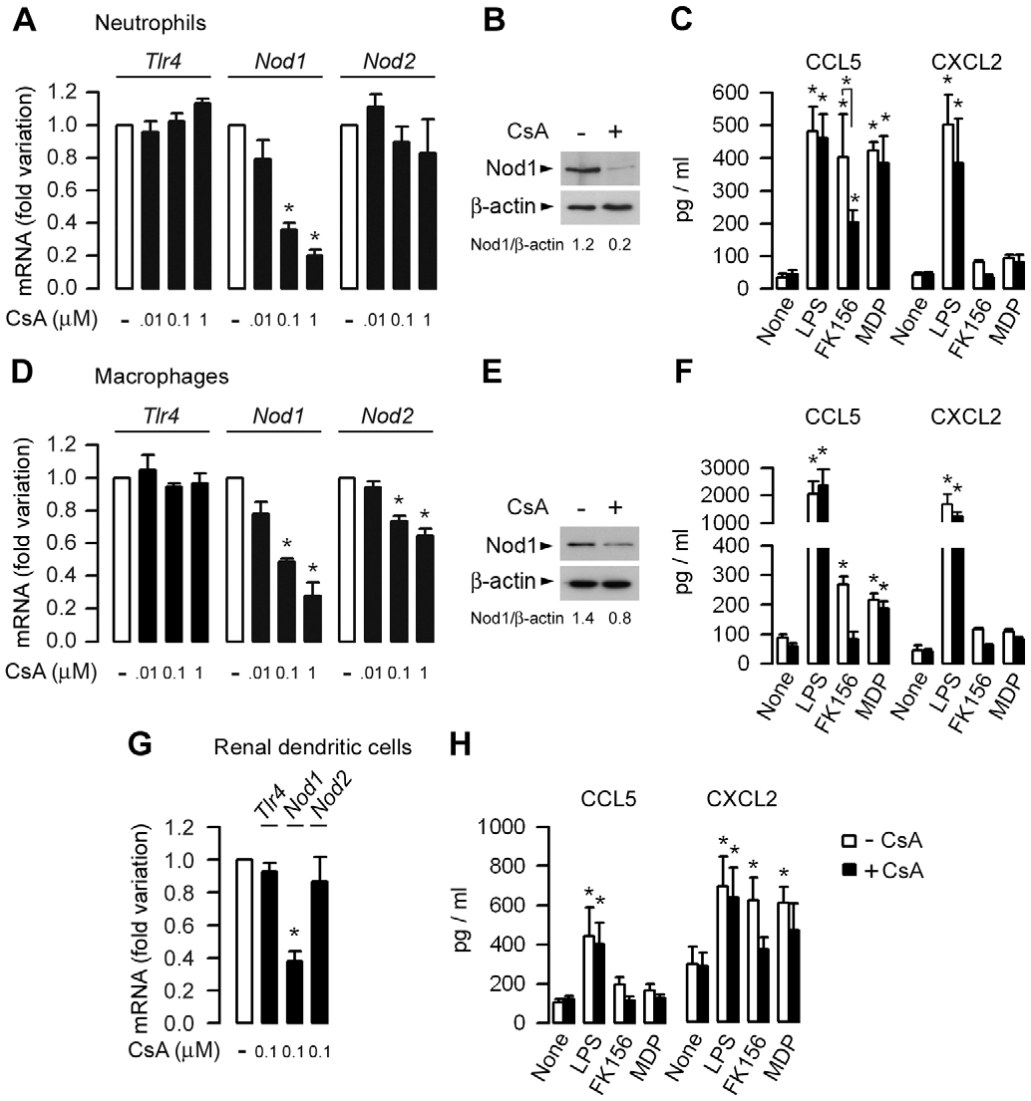
CsA preferentially impairs Nod1 expression in neutrophils, macrophages, and renal dendritic cells. (A, D, G) Fold variations of *Tlr4*, *Nod1* and *Nod2* mRNAs expression measured by quantitative real-time PCR in WT bone marrow neutrophils (A), macrophages (D), and renal CD11c^+^ DCs (G) incubated with CsA for 8 h (neutrophils) or 36–48 h (macrophages, DCs) at 37°C, and compared to corresponding untreated cells. (B and E). Immunodetection of the Nod1 protein in untreated (− CsA) and CsA-treated (+ CsA) neutrophils and BMMs. (C, F, H) Effects of 100 nM CsA on CCL5 and CXCL2 production [mean values of mean individual values (n = 3–4) from 3 to 4 separate experiments] in supernatants from neutrophils (C) and macrophages (F) (10^6^ cells/well, or renal DCs (10^5^ cells/well) sequentially incubated without or with CsA for 8 h (neutrophils) or 36–48 h (macrophages, renal DCs), then with CsA and 10 ng/ml LPS, or 1 µM FK156 or MDP for a further 6 h at 37°C. Values are presented as mean ± SE. *, *p*<0.05 between groups or *versus* untreated or CsA-treated cells (Two-tailed, unpaired Student's *t* test).

We next analyzed the effects of CsA on *Tlr4* and *Nod* mRNAs expression in neutrophils, macrophages, and renal DCs. Incubating primary bone marrow neutrophils with CsA for 8 h or bone marrow macrophages (BMMs) with CsA for longer times (48 h) significantly decreased the relative levels of Nod1 mRNA and protein expressions, and to a lesser extent reduced the expression of *Nod2* mRNA, but not that of *Tlr4* mRNA (Figure 5A, B, D and E). Renal DCs expressing Nod1 and Nod2 [37], were shown to produce substantial amounts of CXCL2, which is involved in the recruitment of neutrophils in the kidneys following UPEC challenge [26], [27], [38]. Incubating highly-enriched CD11c^+^ cells isolated from WT kidneys by gradient centrifugation and magnetic beads separation [39] with 100 nM CsA for 48 h significantly reduced *Nod1* mRNA expression without affecting the expression of *Tlr4* or *Nod2* (Figure 5G). However, the small number of purified renal CD11c^+^ cells obtained did not permit reliable Western blot analysis of the Nod1 protein. Consistent with an inhibitory action of CsA on Nod1, the production of CCL5, which has been shown to be highly sensitive to Nod agonist stimulation [40], when stimulated by the Nod1 agonist FK156 was significantly lower in CsA-treated than in untreated neutrophils and macrophages (Figure 5C and F). CsA only slightly, and non-significantly, reduced the FK156-stimulated production of CCL5 in renal DCs (Figure 5H). Given that CXCL2 plays an essential role in the recruitment of neutrophils, we went on to test the effects of CsA on CXCL2 production in neutrophils, macrophages, and renal DCs. Unlike renal MCD cells, CsA only slightly reduced the CXCL2 production stimulated by LPS in neutrophils, macrophages, and renal DCs (Figure 5C, F and H). Taken as a whole, these findings indicate that CsA globally impairs the functional expression of Nod1 in neutrophils, macrophages, and renal DCs.

Since Nod1 senses a number of invasive Gram-negative bacteria, we tested whether UPEC can directly activate *Nod1* mRNA expression in macrophages and whether CsA impairs the UPEC-induced activation of *Nod1*. Incubating WT BMMs with UPEC for 3 h had almost no stimulatory effect on *Tlr4* mRNA expression, but in contrast induced a significant increase in *Nod1* and *Nod2* mRNAs expression (Figure S5). Pre-incubating BMMs with CsA impairs the increase in *Nod1* mRNA expression, and to a much lesser extent that of *Nod2*, caused by the subsequent incubation with UPEC for additional 3 h (Figure S5). These findings further suggest that CsA preferentially alters the activation of *Nod1* induced by UPEC in phagocytic cells.

### Silencing NFATc1 mRNA expression and the 11R-VIVIT peptide inhibitor of NFATs markedly inhibit *Nod1* mRNA expression in macrophages

**Figure 6:**
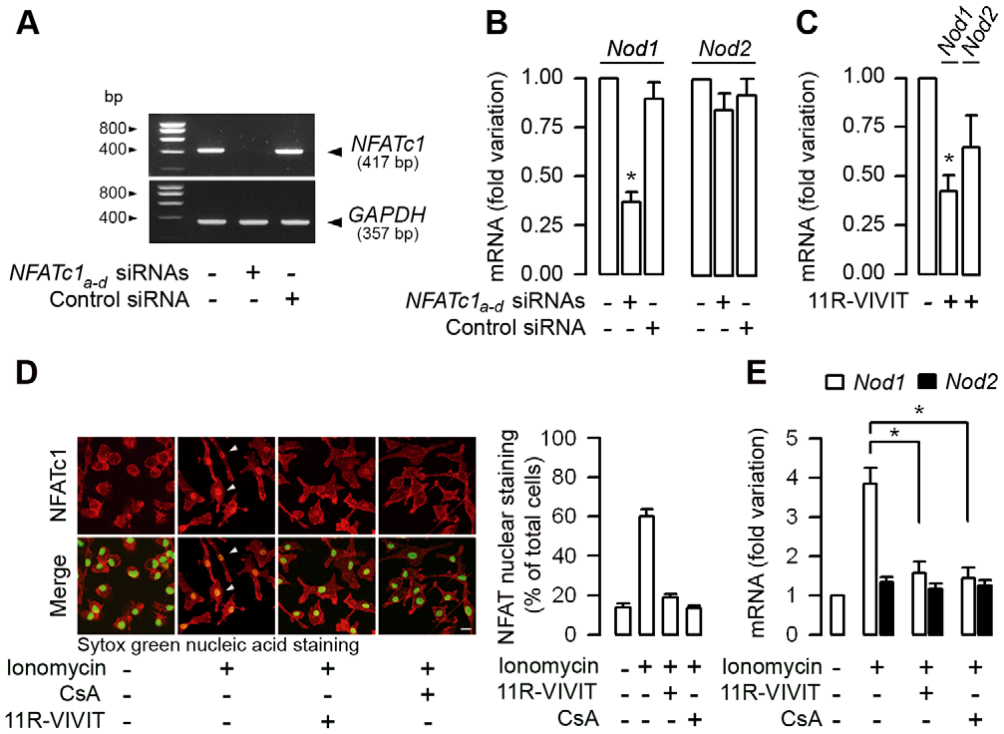
NFATc1 controls *Nod1* mRNA expression in macrophages. (A) Representative reverse transcription-PCR of the inhibition of *NFATc1* in BMMs transfected with a multiple set (n = 4) of *NFATc1_a–d_* siRNAs compared to non-transfected BMMS or with cells transfected with a negative control (Control) siRNA. *GAPDH* mRNA expression was used as internal control. (B and C) Fold variation of *Nod1* and *Nod2* mRNAs measured by quantitative real time PCR in BMMs transfected with the set of *NFATc1_a–d_* siRNAs, or with a negative control (Control) siRNA and compared to non-transfected BMMs (B), and in BMMs incubated with or without 1 µM 11R-VIVIT for 48 h (C) (n = 3 independent experiments in each condition tested). (D) Nucleo-cytoplasmic distribution of the NFATc1 immunostaining (shown in red) (left panel) and percentage of NFATc1 immunostaining co-localized with Sytox green nuclear acid staining (*n* = 7–13 nuclei analyzed for each condition from two separate experiments) (right panel) in WT BMMs sequentially incubated without or with 11R-VIVIT or 10^−7^ M CsA for 48 h, then without or with 2 µM ionomycin for additional 60 min. Bar = 10 µm. (E) Fold variation of *Nod1* and *Nod2* mRNAs expression over corresponding *β-actin* mRNA measured by quantitative real-time PCR in WT BMMs incubated or not with 11R-VIVIT or CsA for 48 h, then with or without 2 µM ionomycin. (*n* = 3 independent experiments). *, *p*<0.05 between groups (Two-tailed, unpaired Student's *t* test).

CsA inhibits the nuclear translocation of NFATs, which in turn inhibit the transcription of T cell effector cytokines [8]. Because CsA preferentially alters Nod1 expression in phagocytic cells, experiments were carried out to test whether the downregulation of the *NFATc1* isoform, which is highly expressed in both murine and human neutrophils and macrophages [41], [42], impairs mRNA expression of Nods. Because neutrophils have a limited life-span, experiments were carried out on mouse BMMs. Knockdown of *NFATc1* mRNA expression using a multiple set of *NFATc1* siRNAs (referred to as *NFATc1_a–d_* siRNA) in WT BMMs resulted in the almost complete inhibition of the expression of *NFATc1* mRNA when compared to non-transfected WT BMMs or cells transfected with a control siRNA (Figure 6A). Silencing *NFATc1* by the set of *NFATc1* siRNAs markedly inhibited the relative level of *Nod1* mRNA expression, but had almost no effect on *Nod2* mRNA expression (Figure 6B). To further assess the inhibitory action of CsA on *Nod* mRNAs expression, experiments were performed on WT BMMs incubated with 11R-VIVIT, a cell-permeable peptide that specifically inhibits the calcineurin-NFATs interaction without affecting calcineurin phosphatase activity [43]. Incubating WT BMMs with 1 µM 11R-VIVIT for 48 h also markedly inhibited the relative levels of *Nod1* mRNA expression, and, to a lesser extent, that of *Nod2* mRNA (Figure 6C). In contrast, knock-down of *NFATc1* mRNA expression or incubation of BMMs with the 11R-VIVIT had no effect on *Tlr4* mRNA expression (Figure S6A and B). Given that inhibition of NFATc1 can affect the transcriptional expression of many proteins, the possibility that *a contrario in vitro* activation of NFATs could specifically stimulate the expression of Nod1 was investigated. NFATs are activated by increased intracellular calcium concentration during T-cell activation [7]. Calcium mobilization induces the dephosphorylation of cytosolic NFATs which translocate into the nucleus [44]. Ionomycin (2 µM, 60 min) induced the translocation of NFATc1 from the cytosol into nuclei from WT BMMs, whereas the pre-incubation of WT BMMs with CsA or 11R-VIVIT totally or almost totally impaired the nuclear translocation of NFATc1 caused by subsequent addition of ionomycin (Figure 6D). Ionomycin also significantly stimulated *Nod1*, but failed to stimulate *Nod2* and *Tlr4* mRNAs expression in WT BMMs compared to untreated cells, or to cells pre-treated with CsA or 11R-VIVIT (Figure 6E and Figure S6C). Collectively, these data strongly suggest a role for NFATc1 as a transcriptional activator of Nod1.

### *In vivo* administration of 11R-VIVIT impairs neutrophil bacterial phagocytic killing capacity of UPEC and increases renal susceptibility to UPEC

**Figure 7:**
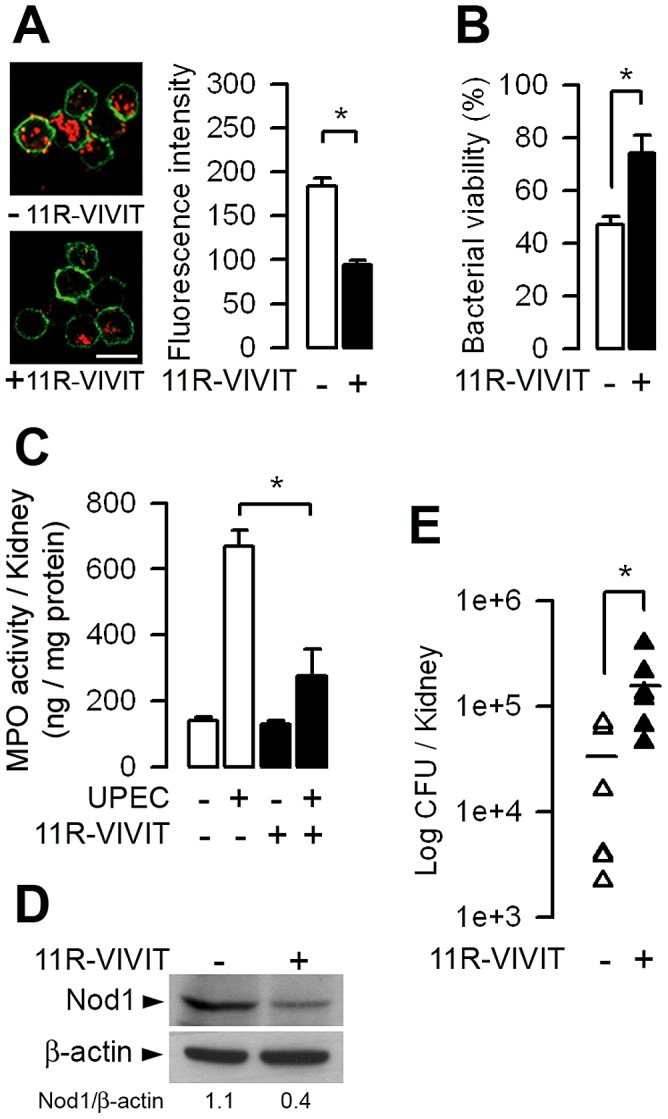
The 11R-VIVIT peptide inhibitor of NFATs decreases bacterial phagocytic killing capacity by neutrophils and increases renal susceptibility to UPEC. WT mice were given daily intraperitoneal injections of 10 µg/kg 11R-VIVIT for 48 h before the transurethral inoculation of UPEC. (A) Illustrations and quantification of internalized Texas red-coupled *E. coli* by peritoneal neutrophils from untreated (−) and 11R-VIVIT-treated (+) mice. Values are represented as mean ± SE of the mean count values (2–4 per condition) from three independent experiments. Cell membranes were stained with CD11b-FITC. Bars = 10 µm. (B) Killing of serum-opsonized *E. coli* by peritoneal neutrophils from untreated and 11R-VIVIT-treated mice. Bacterial viability was expressed relative to control assay performed without neutrophils (*n* = 3–5 determinations from 3 experiments). (C to E) MPO activity (C), representative immunoblot analysis of Nod1 and corresponding ß-actin (D), and bacterial counts (E) in kidneys from untreated and 11R-VIVIT-treated WT mice 24 h after UPEC inoculation. The numbers indicate the ratio of Nod1 over ß-actin densitometric values from 4 experiments and the horizontal bars show the mean of bacterial counts of each group (*n* = 6 determinations in each group). Values are presented as mean ± SE. *, *p*<0.05 between groups (Two-tailed, unpaired Student's *t* test).

Given that the 11R-VIVIT inhibited *Nod1* mRNA expression, 11R-VIVIT should also impair the Nod1-mediated bacterial phagocytic function and decrease the renal defense against UPEC. NPCs isolated from WT mice given daily intraperitoneal injections of 10 µg/kg 11R-VIVIT for 48 h exhibited a significant lower *ex vivo* capacity to internalize *E. coli* and lower phagocytic killing of serum-opsonized UPEC than untreated NPCs (Figure 7A and B). 24 h after the inoculation of UPEC, 11R-VIVIT-treated mice exhibited reduced intrarenal MPO activity and reduced amount of immunodetected Nod1 protein, and significant greater renal bacterial burden than in non-treated WT mice (Figure 7C to E).

### Nod1 stimulating agents partially restore the CsA-induced downregulation of Nod1 and renal resistance to UPEC

**Figure 8:**
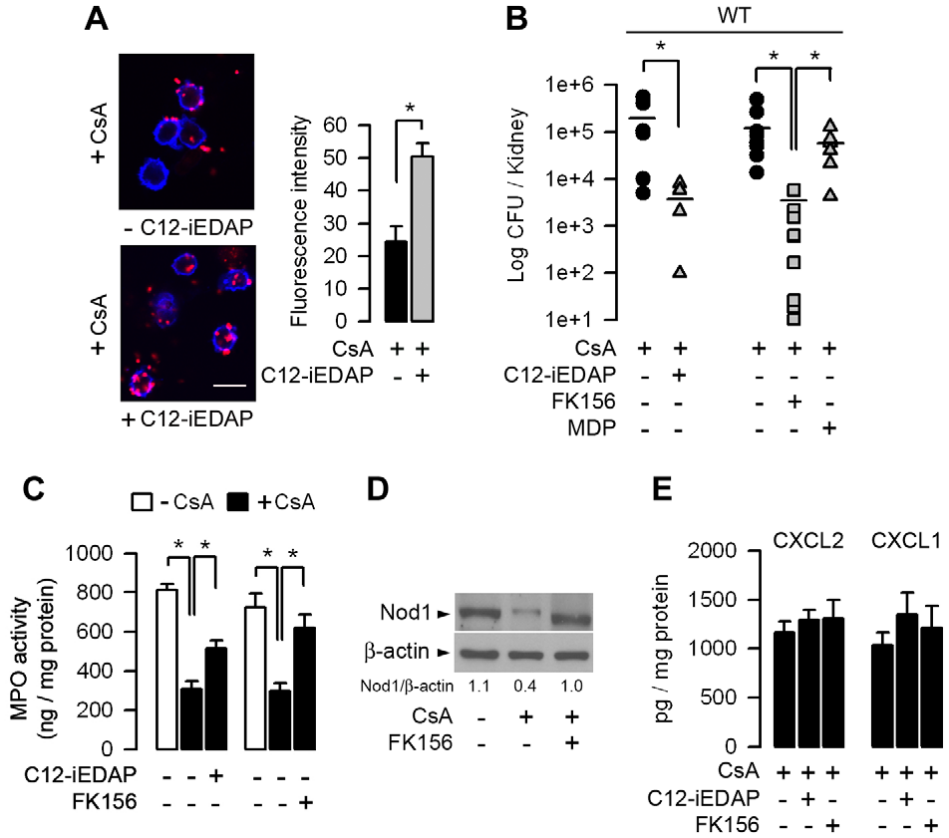
The Nod1 stimulating agonists C12-iEDAP and FK156 stimulate neutrophil bacterial phagocytosis impaired by CsA and increase renal defenses against UPEC. (A) Illustrations and quantification of Texas red-coupled *E. coli* internalized by circulating neutrophils collected from WT mice treated with CsA for 5 days then with CsA plus C12-iEDAP (80 µg per mouse) for a further 5 h. Neutrophils were isolated from the blood of 3 mice under each of the conditions tested. Representative values (mean ± SE) of the mean counts (9–15 per condition) from 3 independent experiments. Cell membranes were stained with WGA Alexa Fluor 647 conjugate. Bars, 10 µm. (B and C) Bacterial counts (B) and MPO activity (C) in kidneys of CsA-treated WT mice pre-treated or not with C12-iEDAP (n = 6), FK156 (n = 9), or MDP (n = 7) (80 µg per mouse), and then challenged 24 h later with *E. coli* HT7. (D) Immunoblot analysis of Nod1 and the corresponding ß-actin in the post-infected kidney homogenates from vehicle- and CsA-treated mice which had received FK156 12 h before UPEC inoculation. (E) Production of CXCL2 and CXCL1 in the 24 h post-infected kidneys from vehicle- or CsA-treated WT mice pre-treated or not with C12-iEDAP or FK156 (n = 6–9 per group). *, *p*<0.05 (A, C, E, Two-tailed, unpaired Student's *t* test; B, Mann-Whitney test).

The fact that the stimulation of Nod1 can enhance systemic innate immunity [20] and that the administration of Nod1 peptide agonists to mice confer resistance against several pathogens [45], led us to test whether the stimulation of Nod1 by synthetic Nod1-stimulating agonists could reinforce renal defense against UPEC. The cell-permeable Nod1 activating agonist C12-iEDAP (50 µg/ml for 24 h) induced a significant increase in *Nod1* mRNA expression, which overcome the inhibition of Nod1 mRNA expression caused by CsA alone (not shown). We then tested whether the *in vivo* administration of synthetic Nod1 agonists can reactivate Nod1-mediated phagocytic function and reinforce renal resistance of CsA-treated mice to UPEC. The capacity of neutrophils to internalize Texas red-coupled *E. coli* was greater in neutrophils from CsA-treated mice that had been treated with C12-iEDAP than in those collected from CsA-treated mice which had not received C12-iEDAP (Figure 8A). Furthermore, intra-peritoneal injection of C12-iEDAP or FK156 (80 µg/mouse) one day before the transurethral inoculation of UPEC to CsA-treated WT mice, induced significant reduction in the renal bacterial burden when compared to CsA-treated mice which had not received C12-iEDAP or FK156, or CsA-treated mice which had received the Nod2 agonist MDP (Figure 8B). The observed decrease in renal bacterial burden was associated with greater MPO activity in the infected kidneys from CsA-treated mice pre-treated with the Nod1 agonists (Figure 8C). Figure 8D illustrates the greater amount of Nod1 protein in the infected kidneys from CsA-treated mice which had been pre-treated with FK156. The amounts of CXCL2 and CXCL1 secreted were not significantly different in the 24-h, post-infected kidneys from CsA-treated mice and the vehicle-treated mice (Figure 8E), suggesting that the administration of Nod1 agonists does not induce any major renal inflammatory response. Collectively, these findings are consistent with the restimulation of Nod1-mediated host protective functions in CsA-treated mice.

### Human transplant recipients treated with CsA exhibit downregulated expression of NOD1

**Figure 9:**
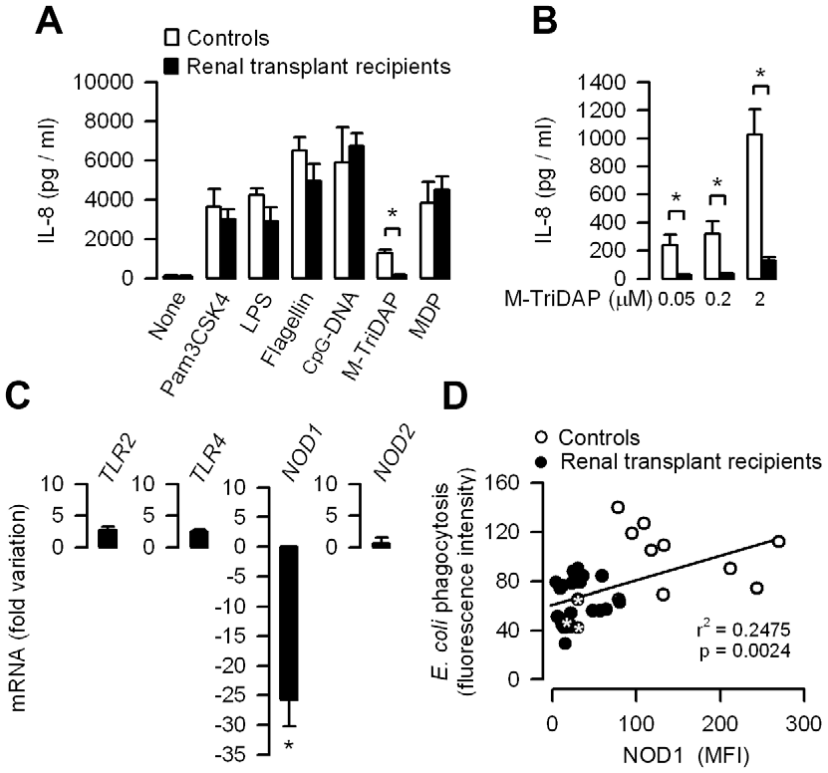
Functional downexpression of NOD1 in leukocytes from CsA-treated renal transplant recipients. (A) IL-8 production in blood samples from healthy volunteers (Controls, n = 10) and renal transplant recipients treated with CsA (n = 25) incubated without (None) or with 1 ng/ml Pam3CSK4 or LPS, 1 µg/ml flagellin, 50 µg/ml CpG-DNA, 1 µM M-TriDAP or MDP. (B) IL-8 production in blood samples from Controls and renal transplant recipients incubated with increasing concentrations of M-TriDAP. (C) Relative fold variation of *TLR2*, *TLR4*, *NOD1* and *NOD2* mRNAs in blood samples from renal transplant recipients (n = 25) compared to that found in Controls (n = 10). (D) Flow cytometry analyses of phagocytosed *E. coli* (expressed as fluorescence intensity of internalized *E. coli*) and mean fluorescence intensity (MFI) of NOD1 expression in neutrophils from healthy volunteers (n = 10) and renal transplant recipients treated with CsA (n = 25). * Transplant recipients who experienced UTI/APN. Values are presented as mean ± SE from n determinations per condition tested. *, *p*<0.05 (A, B, Two-tailed, unpaired *t* test; C, Mann-Whitney test).

Investigations were performed on blood samples from random renal transplant recipients treated with CsA (n = 25) to test whether human renal transplant recipients exhibit similar decrease in NOD1 expression and defective NOD1-mediated bacterial phagocytosis capacity. The demographic characteristics of renal transplant recipients are summarized in Table S1. Transplant recipients all received CsA and additional immunosuppressive drugs, including prednisolone and mycophenolate mofetil, which is a selective inhibitor of the *de novo* synthesis of guanosine nucleotides in T and B lymphocytes [46]. For comparison, investigations were also performed on healthy volunteers (n = 10) used as controls. The production of IL-8 was first measured in whole blood samples incubated with various TLR and NOD agonists. The levels of IL-8 triggered by all TLR agonists tested did not significantly differ in blood samples from transplant recipients and normal healthy controls (Figure 9A). In contrast, the levels of IL-8 production stimulated by the human NOD1 synthetic agonist M-TriDAP was significantly less in blood samples from the transplant recipients treated with CsA than in normal controls (Figure 9A and B). The levels of *NOD1* mRNA, but not those of *NOD2*, *TLR2*, or *TLR4* mRNAs extracted from whole blood samples, were also significantly lower in transplant recipients than in normal controls (Figure 9C). Flow cytometry analysis of phagocytosis of Texas red-coupled *E. coli* and quantification of NOD1 in human neutrophils also revealed that the low levels of NOD1 in the neutrophils from CsA-treated renal transplant recipients were closely correlated with their capacity to phagocyte *E. coli*, which was significantly lower than in the neutrophils from normal healthy controls (Figure 9D). Moreover, three of the transplant recipients analyzed who exhibited low NOD1 expression and low capacity of *E. coli* phagocytosis by neutrophils had a previous history of UTI/APN. These findings suggest that the NFATs-dependent inhibitory mechanism of Nod1-mediated innate immune response identified in the mouse also occurs in human transplant recipients treated with CsA.

## Discussion

In the present work, we show that in mice, CsA reduces renal resistance to the retrograde inoculation of uropathogenic *E. coli*. CsA induces a significant decrease in the production of the chemoattractant chemokines and impairs the recruitment of neutrophils in kidneys from mice infected by UPEC. The primary source (i.e. the epithelial tubule cells or circulating immune cells) of pro-inflammatory mediators produced in experimental models of UTI remains discussed. In accordance with a number of previous studies, medullary collecting duct epithelial cells, which are the first renal tubule cells to come into contact with UPEC during their retrograde ascent, produce substantial amounts of TLR4-mediated CXCL2, which play a key role in the recruitment of neutrophils in the infected kidneys [23], [27], [47]. The fact that CsA impairs LPS-induced production of CXCL2 in renal MCD cells and LPS-induced recruitment of neutrophils, further suggests that the Tlr4-mediated activation of renal epithelial cells contributes to the recruitment of neutrophils in the infected kidneys, at least during the initial phase of infection. Nor can we exclude the possibility that the decrease in *Tlr4* mRNA expression detected in the infected kidneys and in the Tlr4-mediated cell activation detected in murine MCD cells, are due, at least in part, to the cytotoxic action of calcineurin inhibitors [48]. In contrast, CsA had no *in vitro* inhibitory effect on *Tlr4* expression in neutrophils, macrophages, or renal DCs. After being bound to renal collecting duct cells, UPEC induces the rapid recruitment of neutrophils (during the first 6 h), followed by the recruitment of monocytes/macrophages over the next 12–24 h. Although LPS stimulates the *in vitro* production of CXCL2 by neutrophils and inflammatory monocytes/macrophages, these cells do not seem to play major roles in the renal production of chemoattractant chemokines during UPEC infection [38]. Recently, resident renal DCs have been shown to be major source of CXCL2 production, compared to neutrophils and monocytes/macrophages, 20 h following the retrograde inoculation of UPEC [38]. Furthermore, the migration capacity of neutrophils has been reported to be significantly lower in UPEC-infected CD11c deficient mice than in their WT counterparts [38], indicating that CXCL2 production by renal DCs certainly plays some role in the chemoattraction of neutrophils. Here we show that CsA altered *Nod1* mRNA expression in renal DCs, without impairing *Tlr4* mRNA expression and the number of renal DCs in the infected kidneys. Although *in vitro* incubation of renal DCs with CsA did not have much effect on the *in vitro* LPS-induced CXCL2 production, we cannot exclude any *in vivo* participation of renal DCs in the defective migration capacity of neutrophils within infected kidneys from CsA-treated mice.

The present study demonstrated an unexpected effect of CsA on Nod1-mediated neutrophils migration capacity and bacterial phagocytosis. We show both *in vivo* and *in vitro* that CsA impairs Nod1 expression in neutrophils and macrophages. The stimulation of Nod1 by Nod1 stimulating agonist or bacteria was shown to play a role in the recruitment of neutrophils in the intestine [18], [49], and that the number of infiltrating neutrophils was shown to be significantly reduced in injured livers from *Nod1^−/−^* mice challenged with carbon tetrachloride [30]. We also detected defective recruitment of neutrophils in kidneys from *Nod1^−/−^* mice infected by UPEC, and in infected kidneys of WT mice treated with CsA. Given that CsA, which affects TLR4-mediated CXCL2 production in MCD cells and alters the expression of *Nod1* in neutrophils, macrophages, and also renal DCs, these findings suggest that, in addition to impairing the epithelial TLR4-mediated production of chemoattractant chemokines, CsA may also alter the Nod1-mediated capacity of neutrophils to migrate in kidneys infected with UPEC.

Recent studies have highlighted the role of NFAT/calcineurin signaling pathways in controlling innate immunity and in regulating homeostasis of immune cells. Calcineurin/NFATs signaling was shown to negatively regulate myeloid lineage development [50]. The susceptibility to fungal infection caused by CsA was also shown to be the consequence of NFAT-dependent inhibition of an immune innate pathway regulating antifungal resistance in neutrophils. Indeed, Greenblatt et al. [42] reported that the neutrophils of both calcineurin-deficient mice and CsA-treated mice exhibited a defective ability to kill *Candida albicans* without any noticeable changes in the classical fungicidal activity of neutrophils. These authors showed that calcineurin regulates the ability of neutrophils to kill *C. albicans via* another anti-microbial pathway, which involves the C-type lectin-like receptor dectin-1 and IL-10 production. Given that Nod1 and Nod2 are not directly involved in the recognition of *C. albicans* [51], these findings suggest that CsA may affect NFATs-dependent cellular signaling activated by Gram-negative bacteria or fungi in different ways. The NFATc3/c4 isoforms were also shown to be required for TLR-induced innate inflammatory response in monocytes/macrophages [52]. Our results strongly suggest that NFATc1 controls Nod1 at the transcriptional level. *In silico* analysis (*Genomatix* Software GmbH) has identified putative NFAT binding sites in human and murine Nod1 and Nod2 promoter regions. Downexpression of NFATc1 inhibited Nod1 more efficiently than Nod2. However, the *in silico* analysis did not permit us to predict differences in the number of putative binding sites for NFATc1 on the promoter regions of Nod1 and/or Nod2. We have no direct explanation for the preferential inhibitory effect of silencing NFATc1 on Nod1 expression. The fact that much less Nod1 than Nod2 is present in immune cells may account, at least in part, for the greater reduction in *Nod1* mRNA expression induced by *NFATc1* silencing. Nevertheless our findings strongly suggest that CsA, through NFATc1 inhibition, alters Nod1-mediated phagocytic functions. In agreement with this, inhibition of the calcineurin phosphatase activity has been reported to decrease phagocytosis in macrophages [53], further suggesting that NFATc1 is essential for proper activation of the phagocytosis process. Collectively, these findings indicate that NFATs, which play key roles in adaptive T cell functions, are critical cellular mediators of the innate immune responses.

Although the present findings indicate that CsA directly alters Nod1 expression, it may also affect other immune receptors and downstream signaling pathways in various different ways. Calcineurin serine threonine phosphatase downregulates TLR-mediated signaling pathways in macrophages, whereas CsA and its newer counterpart Tacrolimus have been shown to activate NF-κB and induce cytokine expression in inactivated macrophages [54]. It has been also reported that the activation of DCs and macrophages by Tacrolimus can induce a state of reduced responsiveness to LPS [55], similar to the LPS-induced transient state of tolerance observed following a subsequent LPS challenge [56]. During bacterial infection, it seems likely that various TLRs and NLRs are activated in response to the invading pathogen or to microbial components, such as LPS or PGN, released into the bloodstream and in the infected tissues [57]. Moreover, the interplay between Nods and TLRs may be critical for the induction of protective immune responses [14], [58], [59]. Therefore, it is conceivable that altered epithelial TLR4-mediated chemokine production caused by CsA, may potentiate the deleterious inhibitory effects of CsA on Nod1-mediated neutrophil functions, leading to a more pronounced decrease in host resistance to bacterial infection.

Long-term use of immunosuppressive drugs, used to prevent graft rejection, increases the susceptibility of transplant recipients towards bacterial infection [2], [5], [6]. Until recently, the impact of immunosuppressive therapy was considered to be largely non-specific. However, several groups of researchers have reported changes in the numbers and/or functions of circulating leukocytes, including polymorphonuclear neutrophils from transplant patients acquiring infections [21], [22]. Moreover, it has already been suggested that abnormalities in neutrophil functions, including impaired migration capacity following fMLP stimulation, are indicators of sepsis in solid organ transplant recipients [60]. Neutrophils from renal transplant recipients have been reported to exhibit diminished phagocytic activity and reduce bactericidal activity against *Klebsiella pneumoniae*, compared to the activities seen with neutrophils from healthy subjects [61]. *In vitro* studies have also shown that CsA reduces both neutrophil phagocytosis capacity and ROS production [62], [63]. Analysis of a panel of blood leukocyte phenotypes and functions also revealed that transplant recipients (renal and renal/pancreas) most of whom were receiving CsA and subjected to infection exhibited a reduction in ROS production [21]. In the present study, the investigations performed on renal transplant recipients have revealed downregulated expression of NOD1 in circulating leukocytes, similar to that found in CsA-treated mice. The low capacity for *E. coli* phagocytosis appears to be closely correlated with a low expression of NOD1 in the neutrophils of renal transplant recipients. Since Nod1^−/−^ mice are more susceptible than WT to early *Streptococcus pneumoniae* sepsis, and conversely, that PGN recognition by Nod1 enhances killing of *S. pneumoniae* and *Staphylococcus aureus* by neutrophils [19], it is conceivable that the observed downregulation of NOD1 caused by CsA may also impair the capacity of neutrophils from renal transplant recipients to kill Gram-positive bacteria. However, we cannot exclude the possibility that the results from investigations performed in human renal transplant recipients could have been flawed by several confounding factors, such as the concomitant administration of several immunosuppressive drugs and some degree of renal impairment. The consistency with which CsA downregulated NOD1 strongly suggests that the impairment of NOD1-mediated bacterial phagocytic capacity caused by CsA may therefore represent an additional risk factor for the occurrence of UTI/APN in human transplant recipients. Despite antibiotic prophylaxis, the frequency of post-graft UTI/APN still remains relatively high, and increasing the resistance of bacteria to antibiotics may also increase the risk of recurrent episodes of UTI/APN. This raises the question of whether alternative therapeutic strategies could help to reduce the frequency of post-graft UTI/APN. A number of studies have provided convincing evidences that pre-treatment of mice with Nod agonists enhances host protection against sepsis, bacterial infection, viruses, or even parasites [64]. Here we also show that administration of Nod1 agonists can restore efficient renal clearance of UPEC in CsA-treated mice. However, further studies will be required to find out whether the administration of synthetic Nod1 agonists alone or in combination with antibiotics could potentially help to reduce the occurrence of UTI/APN in renal grafts.

In summary, we have identified a hitherto-unknown mechanism of the NFATc1-dependent inhibitory action of the Nod1-mediated innate immune response, which may affect host renal antibacterial defenses against invasive uropathogens, and possibly favor the emergence of bacterial infection in renal transplant recipients receiving long-term CsA treatment.

## Materials and Methods

### Ethics statement

All animal experiments were approved by and conducted in accordance with guidelines of the French Agricultural Office and in compliance with the French and European regulations on Animal Welfare (Service de la protection et Santé animale; Approval Number 75–687, revised 2008) and with Public Health Service recommendations. All the efforts were made to minimize suffering of mice.

Blood samples were obtained from transplant recipients and healthy volunteers after being informed and given oral consent, according to French law for non interventional studies using a leftover or a small additional blood sample (Public Health Code, article L1121-1, revised in May 2009). All samples were anonymized. Human and animal studies were approved by the Institutional Ethics Committee (Comité de Protection des Personnes (CCP #5) affiliated to the Tenon Hospital (AP-HP)-University Paris 6 (Approval CCP-0612/2011). All experiments were conducted in accordance with the principles expressed in the Declaration of Helsinski.

### Renal retrograde infection in mice

Adult female (8–10 week old) WT mice (supplied by the Centre d'Elevage Janvier, Le Genest-Saint-Isle, France), *Rag2^−/−^* mice, and *Nod1* ^−/−^ and *Nod2^−/−^* mice from the same C57BL/6 genetic background were used. Mice were infected with the uropathogenic *E. coli* strain HT7 (10^8^ bacteria in 50 µl sterile PBS) introduced *via* the transurethral route into the bladder as described [4], [25]. 100 µl CsA (Neoral, Novartis International Pharmaceutical Ltd, 15 mg/kg), or its vehicle (castor oil) were administered sub-cutaneously to mice for 5 days before the inoculation of UPEC. Bacterial loads (CFU) in kidneys were determined 24 h after infection by plating. Kidney sections were stained using anti-*E. coli* antibody (Interchim), anti-Ly6-G antibody (BD Biosciences), or aquaporin-2 (AQP-2) as described [23].

### Cultured collecting duct cells

Primary cultures of medullary collecting duct (MCD) isolated from WT, *Nod1^−/−^*, *Nod2^−/−^*, and *Tlr4^−/−^* mice kidneys were grown as described [23]. Experiments were carried out on confluent cells two weeks after seeding.

### Macrophage, neutrophil, and renal dendritic cell isolation

Bone marrow neutrophils and circulating blood neutrophils were isolated by gradient density centrifugation using Ficoll-Paque PREMIUM (GE Healthcare) as described elsewhere [20]. Bone marrow-derived macrophages (BMMs) were isolated and grown as described [40]. Indirect immunofluorescence studies were performed on WT BMMs using a mouse anti-NFATc1 monoclonal antibody (Thermo Scientific Pierce Antibodies) and Sytox green nucleic acid stain (Invitrogen). Renal dendritic CD11c^+^ cells were isolated as previously described with slight modifications [39], [65]. For each cell preparation both kidneys from 5 naïve WT mice were used. Briefly, the kidneys of each mouse were minced and then digested for 45 min at 37°C with 1 mg/ml collagenase (Roche Diagnostics, Meylan, France) and 10 µg/ml DNAse I in RPMI 1640-Glutamax medium (Life Technologies) supplemented with 10% heat-inactivated fetal calf serum, 10 mM HEPES, 100 U penicillin, and 0.1 mg/ml streptomycin. Kidney homogenates from each mouse were then filtered through 70 µm nylon mesh, washed with PBS, centrifuged (250 g, 5 min), resuspended in 3 ml of 0.01 M ethylenediaminetetracetic acid (EDTA) in FCS and layered on top of 3 ml Histopaque-1077 (Sigma). Density centrifugation (400 *g*, 30 min) was performed at room temperature. The interphase cells were then harvested, washed, and resuspended in 600 µl MACS buffer (Miltenyi Biotec.). CD11c^+^ cells were then isolated using microbead-labeled specific monoclonal antibody (clone N418, Miltenyi Biotec.), and separated using magnetic beads according to the manufacturer's instructions. The enriched- CD11c^+^ cell suspension obtained from 5 mice were then pooled and used for the cytokine assay.

### *In vitro* neutrophil migration assay

The migration capacity of neutrophils isolated from untreated or CsA-treated WT mice or *Nod1^−/−^* mice was analyzed using the Boyden chamber technique as previously described [30]. After the lysis of red blood cells, blood samples from vehicle- and CsA-treated WT were laid on the top of a Ficoll-Paque PREMIUM (GE Healthcare, Uppsala, Sweden) density gradient, then centrifuged (400 g, 30 min at 4°C), and the bottom layer containing the neutrophil-enriched fraction was collected. 10^6^ neutrophils were then resuspended in 200 µl Hank's buffered salt solution (HBSS) containing 0.5% bovine serum albumin and added to the upper compartment of a Transwell Clear membrane insert (3 µm pore size, Corning Inc., Lowel, MA). The lower compartment (600 µl) of the chamber contained either HBSS alone or supplemented with fMLP (10^−7^ M) or CXCL2 (200 ng/ml). Incubations were performed at 37°C for 40 min in a 5% CO_2_/95% air atmosphere. A neutrophil migration assay was also carried out using isolated WT and *Tlr4^−/−^* MCDs seeded and grown to confluence in defined DMEM/Hams'F12 culture medium [23] on the apical side of the filters. Confluent WT MCD cells were then incubated with or without 100 ng/ml CsA for 48 h, then with or without (10 ng/ml) LPS, which was added when required to the upper compartment of the chamber for 4 h in a 5% CO_2_/95% air atmosphere. The lower compartment contained 10^6^ WT neutrophils resuspended in 600 µl defined culture medium. In all cases, the filters were rinsed, then fixed in methanol and stained using the RAL 555 Kit (Réactifs RAL, Martillac, France). The neutrophils (stained deep purple) detected in the filters were counted by microscopic observation. MCD cells (stained pale red) were also stained using the RAL kit containing eosin. In parallel, the transepithelial electrical resistance (R_T_) was measured using dual silver/silver chloride (Ag/AgCl) electrodes connected to a Millicel-ERS voltohmmeter (Millipore, Billerica, MA).

### *E. coli* phagocytosis and bacterial killing assay

Enriched-neutrophil peritoneal cells collected by peritoneal lavages 3 h after a single intraperitoneal injection of 1.5 ml of thioglycollate (Bio-Rad Laboratories) were incubated with Texas red-coupled *E. coli* (10^4^ bacteria/10^7^ cells) for 30 min at 37°C, and then stained with CD11b-FITC or Wheat Germ Agglutinin (WGA)-Alexa Fluor 647 (Invitrogen) to delineate cell peripheries. The internalization of *E. coli* was determined by measuring the intracellular red fluorescence intensity using confocal microscopy analysis as described elsewhere [66]. For the *ex vivo* bacterial killing assay, *E. coli* were mixed without or with peritoneal neutrophils (10^3^ bacteria/10^6^ neutrophils) following the same procedure as described elsewhere [20].

### Renal transplant recipients

Blood samples from 25 renal transplant recipients with a functioning graft during the first three years after surgery and exposed to CsA (Table S1) were randomly taken during the regular routine consultations at Tenon hospital (Assistance Publique-Hôpitaux de Paris, France). In all cases, the blood samples were taken at least 6 months after surgery. In addition, ten healthy volunteers served as normal controls.

### Real-time PCR and reverse transcription PCR

Total RNA from mouse kidneys, neutrophils, or macrophages was purified with RNAble (Eurobio laboratories) and reverse transcribed using Moloney Murine Leukemia Virus reverse transcriptase (Invitrogen). cDNA was subjected to quantitative real-time PCR using a Chromo IV sequence detector (MJ Research). The mouse *Tlr2*, *Tlr4*, *Nod1*, *Nod2* and *ß-actin* primers used and the corresponding Taqman probes are listed in Table S2. PCR data were reported as the relative increase in mRNA transcripts *versus* that found in uninfected kidneys or vehicle-treated neutrophils or macrophages cells and corrected using the respective levels of ß-actin mRNA. Quantitative real-time PCR was also performed on RNA extracted from blood samples of renal transplant recipients using human *TLR2*, *TLR4*, *NOD1*, *NOD2*, and *ß-ACTIN* primers and corresponding Taqman probes (listed in Table S2). PCR data were reported as the relative increase in mRNA transcripts *versus* that found in a pool of RNA of untreated leukocytes from healthy volunteers. For reverse transcription PCR, cDNA and non-reverse transcribed RNA (250 ng) from cultured mouse MCD cells or BMMs were amplified for 35 cycles in 35 µl of Platinum Blue PCR SuperMix (Invitrogen) containing 10 pmol of mouse *NFATc1*, *Tlr4*, *Nod1*, *Nod2*, or *GAPDH* primers (described in Table S2). Amplification products were run on a 2% agarose gel containing SYBR Safe DNA gel stain (Invitrogen) and photographed.

### Small interfering RNA

Experiments were performed using different predesigned HP GenomeWide (Qiagen, Courtaboeuf, France) siRNAs (referred to as NFATc1a, b, c, and d) for the murine *NFATc1* gene target DNA sequence. NFATc1_a_ DNA sequence: 5′-TCGGATCGAGGTGCAGCCCAA-3′; sense: 5′-GGAUCGAGGUGCAGCCCAATT-3′; antisense: 5′-UUGGGCUGCACCUCGAUCCGA-3′; NFATc1_b_ DNA sequence: 5′-CACGGTTACTTGGAGAATGAA-3′; sense: 5′-CGGUUACUUGGAGAAUGAATT-3′; antisense: 5′-UUCAUUCUCCAAGUAACCGTG-3′; NFATc1_c_ DNA sequence: 5′-CCCGTCCAAGTCAGTTTCTAT-3′; sense: 5′-CGUCCAAGUCAGUUUCUAUTT-3′; antisense: 5′-AUAGAAACUGACUUGGACGGG-3′; NFATc1_d_ DNA sequence: 5′-CCGGGACCTGTGCAAGCCAAA-3′; sense: 5′-GGGACCUGUGCAAGCCAAATT-3′; antisense: 5′-UUUGGCUUGCACAGGUCCCGG-3′. A universal negative control siRNA (target DNA sequence: 5′-AATTCTCCGAACGTGTCACGT-3′; sense: 5′-UUCUCCGAACGUGUCACGUdTdT-3′; antisense: 5′ ACGUGACACGUUCGGAGAAdTdT-3′) was also used (Qiagen). Single strand sense and antisense RNA nucleotides were annealed to generate a RNA duplex according to the Manufacturer's instructions. WT BMMs were seeded in 6-well plates and incubated with 10 nM of each siRNA tested and 2 µl of Lipofectamine RNAiMAX Reagent (Invitrogen) for 48 h at 37°C before use. As a control, we checked that each of the NFATc1 (a to d) siRNAs inhibited *NFATc1* mRNA expression in macrophages using reverse-transcription PCR (not shown).

### Western blotting

Mouse kidney homogenates and BMMs were lysed and processed for Western blotting using mouse anti-TLR4 [25]), anti-Nod1 (Cell Signaling) or anti-Nod2 (eBiosciences) antibodies, and phospho-RIP-2 (Ser 176), and total RIP-2 (Ozyme) antibodies. Protein bands were revealed using horse raddish peroxidase-conjugated goat anti-rabbit IgG (Jackson Immunoresearch), and analyzed by Western Blotting.

### ELISA

Cytokine production was measured in mouse kidney homogenates, or cell supernatants using DuoSet mouse ELISA kits (R&D Systems, Minneapolis, MN). Neutrophils, macrophages, or cultured MCD cells were incubated either with LPS (*Escherichia coli* 0111:B4 LPS Ultra-Pure, InvivoGen, Toulouse, France), 50 µg/ml C12-iEDAP (InvivoGen), 1 µM FK156 (provided by Nami Kawano, Astellas Pharma Inc., Osaka, Japan), or 1 µM MDP (InvivoGen, Toulouse, France) for 8 to 18 h at 37°C. For FK156 and MDP stimulations, mouse macrophages were pre-treated with 1 µM cytochalasin D (Calbiochem, Darmstadt, Germany) for 30 min to allow efficient internalization of the synthetic Nod activating agonists as described elsewhere [40]. Human blood samples (10 µl) were incubated in 500 µl RPMI culture medium (Invitrogen) at 37°C alone or with 1 ng/ml Pam3CSK4 (InvivoGen) or LPS, 1 µg/ml flagellin (InvivoGen); 50 µg/ml unmethylated CpG-DNA (HyCult Biotechnology, Uden, The Netherlands), 50 nM MDP or various concentrations (0.05–2 µM) of M-TriDAP (InvivoGen) for 18 h. IL-8 production was measured using a DuoSet human ELISA kit (R&D Systems, Lille, France). All the reagents used were tested negative for endotoxin contamination using the Limulus amoebocyte assay according to the Manufacturer's recommendations (QCL-1000, Biowhittaker, Buckinghamshire, UK). MPO activity was measured using HyCult Biotechnology ELISA kit.

### Flow cytometry analysis

The cell populations infiltrating the infected mouse kidneys were analyzed by flow cytometry. 24 h after UPEC infection, kidneys were carefully rinsed with PBS to remove the remaining circulating blood cells. The kidneys were then minced and digested for 45 min at 37°C with 1 mg/ml collagenase (Roche Diagnostics) and 10 µg/ml DNAse I in the same RPMI 1640-Glutamax medium (Life Technologies) as described above for the isolation of renal DCs. After rinsing, kidney homogenates were then passed through a 70 µm pore sized nylon Cell Strainer (BD Biosciences) with 15 ml PBS. The resulting cell suspension was centrifuged (1600 rpm, 10 min) again and then resuspended (10×10^6^ total cells/ml) in FACS buffer containing 2% BSA and 0.05% sodium azide. Non-specific binding of antibody to Fc receptors was blocked by incubating the cell suspension with the anti-mouse CD16/32 (2.4G2) antibody (10 µg/ml) and ChromePure rat IgG (100 µg/ml, Jackson Immunoresearch) for 30 min at 4°C. Cells were then incubated in pre-determined optimal concentrations of fluorochrome-conjugated antibodies to cell surface antigens or matching isotype control antibodies for 30 min at 4°C. APC anti-mouse Ly-6G/Ly-6C (Gr1; RB6-8C5), Pe anti-mouse F4/80 (BM8), PerCP/Cy5.5 anti-mouse CD11b (M1/70), Pacific Blue anti-mouse (MHC-II/IA/IE, M5/114.15.2) and PeCy7 anti-mouse CD45 (RA3-6B2), and matching fluorophores-conjugated antibodies isotypes were purchased from Biolegend. PeCy7 anti-mouse CD11c (HL3) and V500 anti mouse CD45.2 (104) were purchased from BD Pharmingen. Fluorescent measurements were conducted with identical settings on at least 100,000 CD45^+^ cells per kidney per experiment using a BD FACSCanto II cytometer operating BD FACSDiva software v6.1.3 (BD Biosciences, Erembodegem, Belgium), and FlowJo v7.6.5 (Tree Star. Inc., Ashland, OR). Analyses of NOD1 expression and bacterial phagocytosis capacity by human neutrophils were also analyzed by flow cytometry. Human whole blood samples (1 ml) were incubated with Texas red-coupled *E. coli* (10^7^ bacteria) (Molecular Probes) with gentle stirring for 30 min at 37°C, while negative control samples were kept on ice before analysis. Red blood cells were then lyzed by adding 10 ml NH_4_Cl 0.8% wt/vol for 15 min. Trypan blue (0.05 mg/ml) was added to the samples to reduce the quenching of surface-bound fluorescence. Samples were centrifuged (400 g, 10 min) at 4°C to remove cell debris, and pelleted leukocytes were then rinsed in PBS. In parallel, aliquots of NH_4_Cl-treated blood samples were permeabilized with methanol, and then incubated with an anti-human NOD1 antibody (Imgenex Corp.). All fluorescence measurements were conducted with identical settings and forward and side-scatter parameters to identify the neutrophil population and to gate out other cells and debris [67].

### Statistical analysis

Statistical analysis was performed using the GraphPad Prism program. The unpaired *t* test, (two-tailed *p* values) was used to compare two groups. The distribution of three or more groups was analyzed by One-Way ANOVA and the Kruskal-Wallis test. The Mann-Whitney test was used to compare the group with one another. A *p* value<0.05 was considered significant.

### Accession numbers

The mouse gene accession numbers (GenBank) are as follows: ß-actin, NM_007393.3; GAPDH, AK144690; NFATc1/NFAT2, NM_016791.4; Nod1, NM_172729; Nod2, NM_145857; RipK2, NM_138952.3; Tlr2, NM_011905.3; Tlr4, NM_021297, Tlr5, AF186107.1; Tlr9, AF314224.

The human gene accession numbers (GenBank) are as follows: GAPDH, NM_002046.3; NOD1, NM_006092.2; NOD2, NM_022162.1; TLR2, NM_003264.3; TLR4, NM_138554.3.

The mouse protein accession number (UniProtKB/Swiss-Prot) are as follows: ß-actin, P60710; CCL5, P30882; CD11b/integrin alpha-M/beta-2; P05555; CXCL1; P12850; CXCL2, P10889; NFATc1/NFAT2, 088942; Nod1, Q8BHB0; Nod2, Q8K3Z0; RipK2, P58801; Tlr2, Q9QUN7; Tlr4, Q9QUK6, Tlr5, Q9JLF7; Tlr9, Q9EQU3.

The human protein accession numbers (UniProtKB/Swiss-Prot) are as follows: IL-8, P10145; NOD1, Q9Y239.

## Supporting Information

Figure S1 Identification of the immune cell populations in kidneys from vehicle- and CsA-treated mice 24 h after the retrograde inoculation of UPEC. (A and B) Strategy of gating and representative illustrations of flow cytometry dot-plots identifying the main CD45^+^ cell types present in the 24 h post-infected kidneys from vehicle-treated (A) and 5-days CsA-treated (B) WT mice. F4/80^+^ CD11b^+^ Gr1^INT^ MHC-II^−^ CD11c^−^ inflammatory monocytes/macrophages, F4/80^+^ CD11b^LO^ Gr1^−/LO^ MHC-II^+^ CD11c^+^ renal dendritic cells (DCs), and F4/80^−^ CD11b^+^ Gr1^HI^ MHC-II^−^ CD11c^−^ polymorphonuclear neutrophils (PMNs) were identified as indicated. (C) The histograms indicate the percentage of PMNs, inflammatory monocytes/macrophages, and DCs out of the total renal cells in the 24 h post-infected kidneys from vehicle- and CsA-treated mice (n = 3 in each group). Values are presented as mean ± SE. *, *p*<0.05 (Two-tailed, unpaired Student's *t* test).
(TIF)

Figure S2 Migration capacity of neutrophils isolated from CsA-treated WT mice and *Nod1^−/−^* mice. (A–D) Circulating neutrophils isolated from CsA- and vehicle-treated WT mice (A and B) or naive WT and *Nod1^−/−^* mice (C and D) were placed in the upper compartment of a Boyden chamber and the basal compartment was filled with HBSS medium alone (unstimulated condition, None) or supplemented with 10^−7^ M fMLP or 200 ng/ml CXCL2. (A and C) Illustrations of the difference in the quantity of neutrophils attracted in the filters (shown in red, arrowheads). (B and D) Quantification of the number of neutrophils attracted per filter surface area after 40 min incubation at 37°C without or with addition of fMLP (B and D) or CXCL2 (B) to the basal compartment. Values are represented as mean ± SE from mean count values (2–4 individual filters per condition) from 3 independent experiments. *, *p*<0.05 (Two-tailed, unpaired Student's *t* test).
(TIF)

Figure S3 The phagocytic killing capacities of UPEC are impaired in *Nod1^−/−^* neutrophils. (A and B) Illustration (A) and quantification (B) of Texas red-coupled *E. coli* internalized by peritoneal neutrophils obtained from WT, *Nod1^−/−^*, and *Nod2^−/−^* mice. Values are represented as the mean ± SE of mean count values (3–6 individual filters per condition) from 4 independent experiments. CD11b-FITC was used to delineate cell peripheries. Bar = 10 µm. (C) Killing of serum-opsonized *E. coli* by WT, *Nod1^−/−^*, and *Nod2^−/−^* peritoneal neutrophils. Bacterial viability (*n* = 3–4 determinations from 4 separate experiments per group) was expressed relative to control assay performed without neutrophils. Values are presented as mean ± SE. *, *p*<0.05 (Mann-Whitney test).
(TIF)

Figure S4 CsA impairs the neutrophil migration capacity triggered by medullary collecting duct cells activated by LPS. (A) WT and *Tlr4^−/−^* MCDs cells seeded and grown until confluence on the apical side of semi-permeable filters formed confluent layers of tight epithelial cells (R_T_ ranging between 4400 and 4800 Ω.cm^2^). WT MCD cells were then incubated with or without 100 ng/ml CsA for a further 48 h. LPS (10 ng/ml) was added to the upper compartment of the chamber for 4 h in a 5% CO_2_/95% air atmosphere. The lower compartment contained 10^6^ WT neutrophils resuspended in 600 µl defined culture medium. (B) Illustrations showing the number of neutrophils attracted into the filters (shown in red, arrowheads). (C) Quantification of the number of neutrophils attracted per filter surface area after incubating for 40 min at 37°C with or without addition of LPS to the apical compartment. Values are presented as mean ± SE from mean count values (2–3 individual filters per condition) from 3–4 independent experiments in each group. *, *p*<0.05 (Two-tailed, unpaired Student's *t* test).
(TIF)

Figure S5 UPEC activate *Nod1* mRNA expression in macrophages. *Tlr4*, *Nod1* and *Nod2* mRNA expression in WT BMMs pre-incubated without or with 100 nM CsA for 48 h, then with or without UPEC HT7 isolates (5×10^2^ bacteria) for additional 3 h (*n* = 3–5 individual values from 3 separate experiments). Values are presented as mean ± SE. *, *p*<0.05 (Two-tailed, unpaired Student's *t* test).
(TIF)

Figure S6 Downexpression of *NFATc1* mRNA and NFAT inhibition by 11R-VIVIT do not affect *Tlr4* mRNA expression in macrophages. (A) Fold variations of *NFATc1* and *Tlr4* mRNAs measured by quantitative real time PCR in BMMs transfected with the *NFATc1_a_* siRNA, or with a negative control (Control) siRNA and compared to non-transfected BMMs. (B and C) Fold variation of *Tlr4* mRNA expression measured by quantitative real-time PCR in WT BMMs incubated with or without 1 µM 11R-VIVIT (B and C) or without or with CsA for 48 h, then with or without 2 µM ionomycin for additional 2 h (C). Values are presented as mean ± SE from 3 independent experiments. *, *p*<0.05 (Two-tailed, unpaired Student's *t* test).
(TIF)

Table S1 Demographic characteristics and biological parameters of the renal transplant recipients analyzed.
(DOC)

Table S2 Mouse and human primers and Taqman probes used for quantitative real-time PCR and reverse transcription PCR.
(DOC)
