# Supplementary figures and images for: Cyclosporine A Impairs Nucleotide Binding Oligomerization Domain (Nod1)-Mediated Innate Antibacterial Renal Defenses in Mice and Human Transplant Recipients
Source: PLoS Pathog. 2013 Jan 31;9(1):e1003152. doi: 10.1371/journal.ppat.1003152 (PMC3561241; doi:10.1371/journal.ppat.1003152)

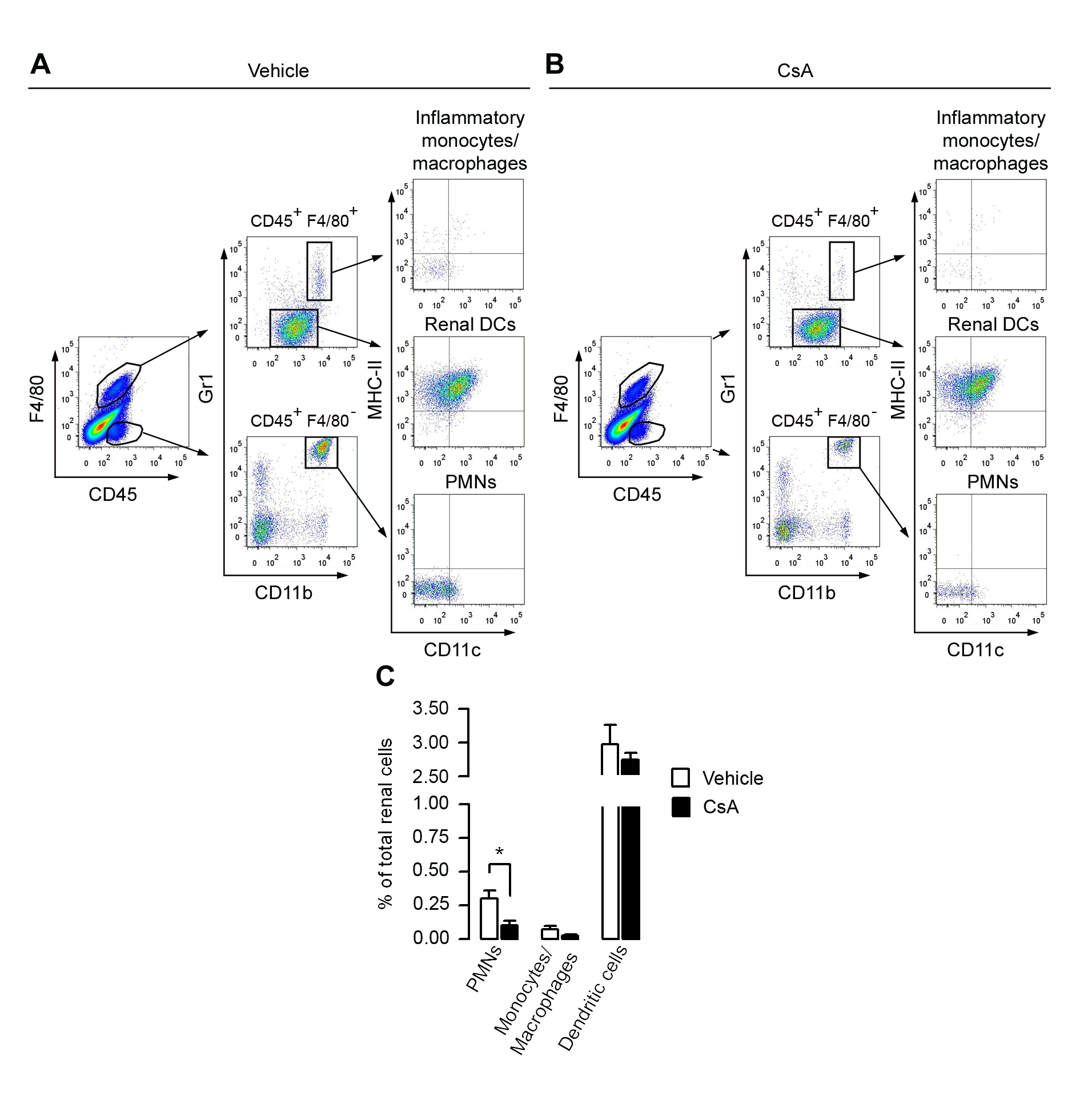

Supplement: Figure S1 — Identification of the immune cell populations in kidneys from vehicle- and CsA-treated mice 24 h after the retrograde inoculation of UPEC. (A and B) Strategy of gating and representative illustrations of flow cytometry dot-plots identifying the main CD45+ cell types present in the 24 h post-infected kidneys from vehicle-treated (A) and 5-days CsA-treated (B) WT mice. F4/80+ CD11b+ Gr1INT MHC-II− CD11c− inflammatory monocytes/macrophages, F4/80+ CD11bLO Gr1−/LO MHC-II+ CD11c+ renal dendritic cells (DCs), and F4/80− CD11b+ Gr1HI MHC-II− CD11c− polymorphonuclear neutrophils (PMNs) were identified as indicated. (C) The histograms indicate the percentage of PMNs, inflammatory monocytes/macrophages, and DCs out of the total renal cells in the 24 h post-infected kidneys from vehicle- and CsA-treated mice (n = 3 in each group). Values are presented as mean ± SE. *, p<0.05 (Two-tailed, unpaired Student's t test). (TIF) [file ppat.1003152.s001.tif]

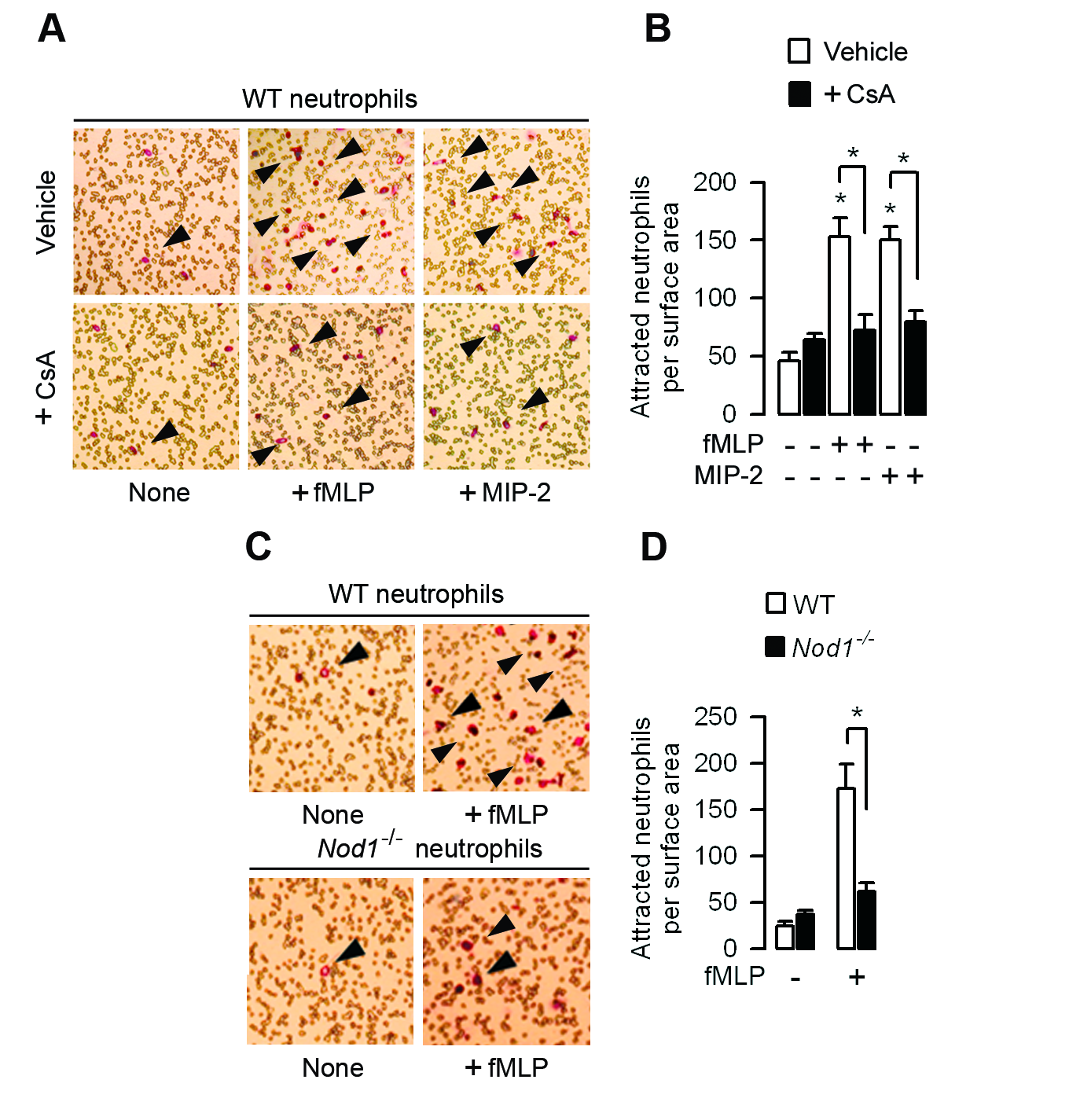

Supplement: Figure S2 — Migration capacity of neutrophils isolated from CsA-treated WT mice and Nod1−/− mice. (A–D) Circulating neutrophils isolated from CsA- and vehicle-treated WT mice (A and B) or naive WT and Nod1−/− mice (C and D) were placed in the upper compartment of a Boyden chamber and the basal compartment was filled with HBSS medium alone (unstimulated condition, None) or supplemented with 10−7 M fMLP or 200 ng/ml CXCL2. (A and C) Illustrations of the difference in the quantity of neutrophils attracted in the filters (shown in red, arrowheads). (B and D) Quantification of the number of neutrophils attracted per filter surface area after 40 min incubation at 37°C without or with addition of fMLP (B and D) or CXCL2 (B) to the basal compartment. Values are represented as mean ± SE from mean count values (2–4 individual filters per condition) from 3 independent experiments. *, p<0.05 (Two-tailed, unpaired Student's t test). (TIF) [file ppat.1003152.s002.tif]

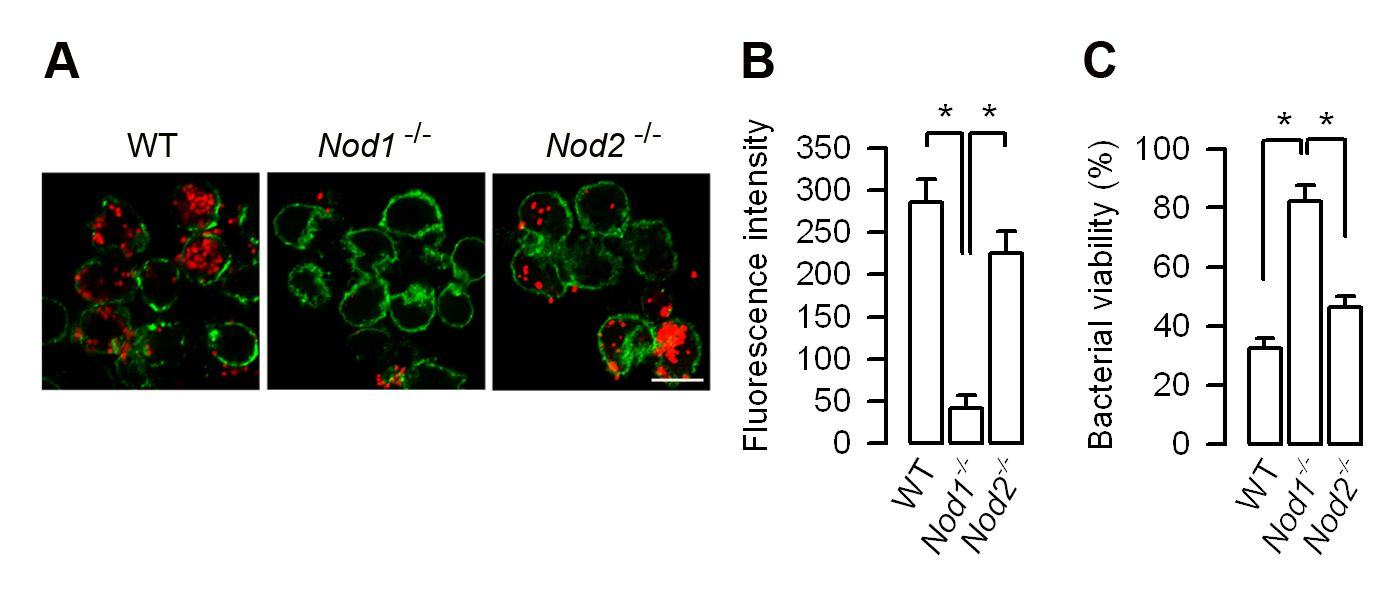

Supplement: Figure S3 — The phagocytic killing capacities of UPEC are impaired in Nod1−/− neutrophils. (A and B) Illustration (A) and quantification (B) of Texas red-coupled E. coli internalized by peritoneal neutrophils obtained from WT, Nod1−/−, and Nod2−/− mice. Values are represented as the mean ± SE of mean count values (3–6 individual filters per condition) from 4 independent experiments. CD11b-FITC was used to delineate cell peripheries. Bar = 10 µm. (C) Killing of serum-opsonized E. coli by WT, Nod1−/−, and Nod2−/− peritoneal neutrophils. Bacterial viability (n = 3–4 determinations from 4 separate experiments per group) was expressed relative to control assay performed without neutrophils. Values are presented as mean ± SE. *, p<0.05 (Mann-Whitney test). (TIF) [file ppat.1003152.s003.tif]

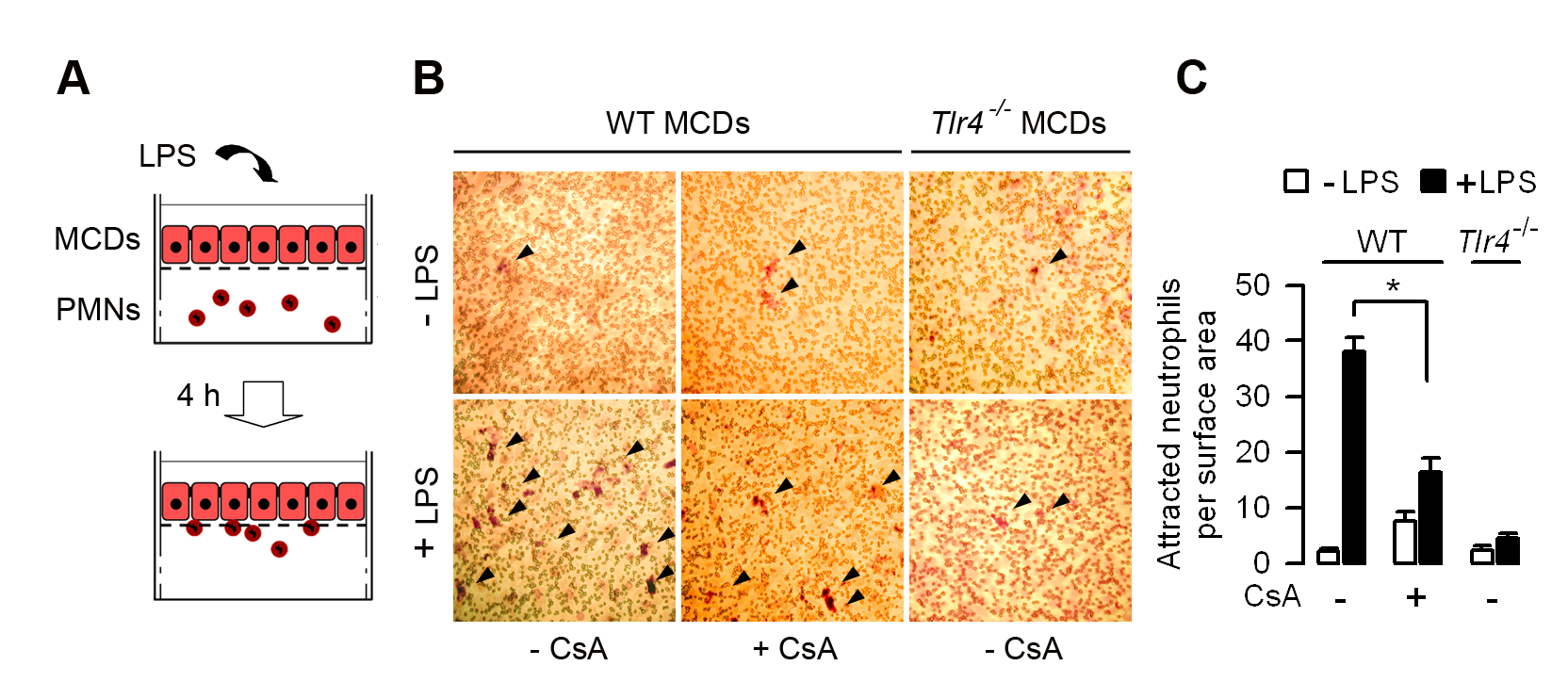

Supplement: Figure S4 — CsA impairs the neutrophil migration capacity triggered by medullary collecting duct cells activated by LPS. (A) WT and Tlr4−/− MCDs cells seeded and grown until confluence on the apical side of semi-permeable filters formed confluent layers of tight epithelial cells (RT ranging between 4400 and 4800 Ω.cm2). WT MCD cells were then incubated with or without 100 ng/ml CsA for a further 48 h. LPS (10 ng/ml) was added to the upper compartment of the chamber for 4 h in a 5% CO2/95% air atmosphere. The lower compartment contained 106 WT neutrophils resuspended in 600 µl defined culture medium. (B) Illustrations showing the number of neutrophils attracted into the filters (shown in red, arrowheads). (C) Quantification of the number of neutrophils attracted per filter surface area after incubating for 40 min at 37°C with or without addition of LPS to the apical compartment. Values are presented as mean ± SE from mean count values (2–3 individual filters per condition) from 3–4 independent experiments in each group. *, p<0.05 (Two-tailed, unpaired Student's t test). (TIF) [file ppat.1003152.s004.tif]

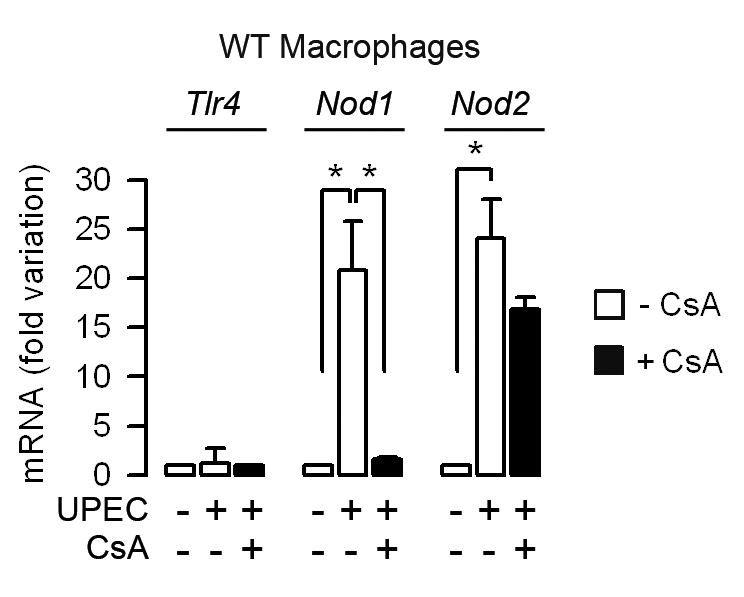

Supplement: Figure S5 — UPEC activate Nod1 mRNA expression in macrophages. Tlr4, Nod1 and Nod2 mRNA expression in WT BMMs pre-incubated without or with 100 nM CsA for 48 h, then with or without UPEC HT7 isolates (5×10^2^ bacteria) for additional 3 h (n = 3–5 individual values from 3 separate experiments). Values are presented as mean ± SE. *, p<0.05 (Two-tailed, unpaired Student's t test). (TIF) [file ppat.1003152.s005.tif]

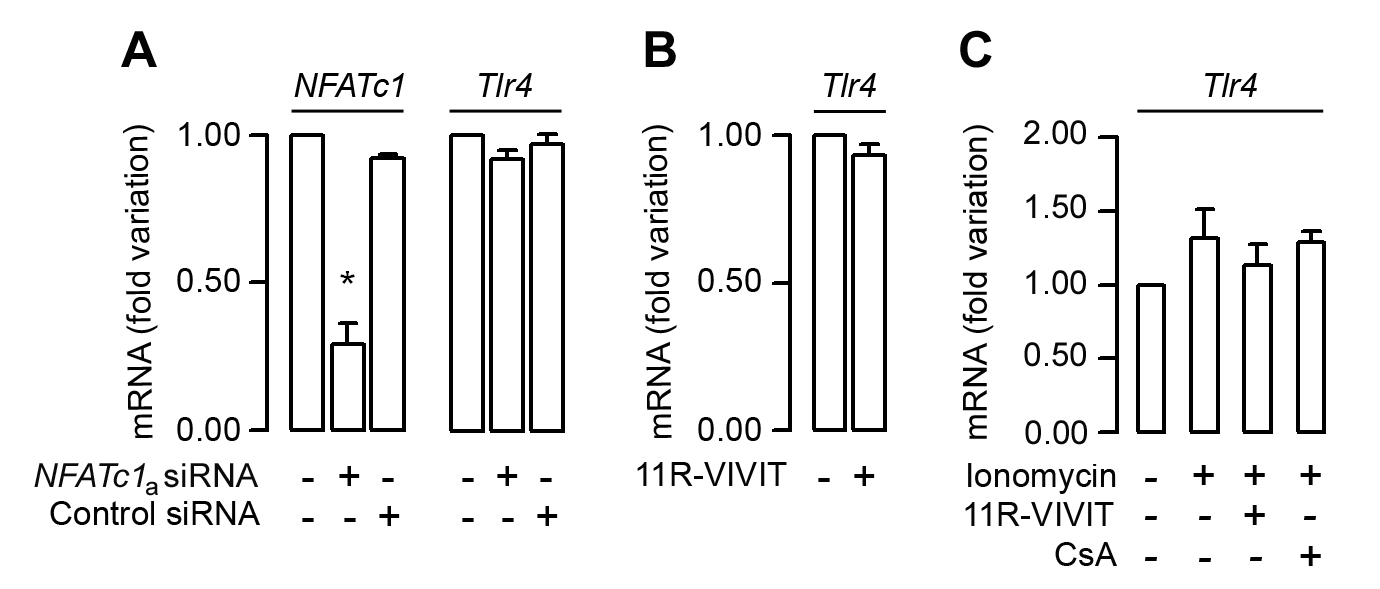

Supplement: Figure S6 — Downexpression of NFATc1 mRNA and NFAT inhibition by 11R-VIVIT do not affect Tlr4 mRNA expression in macrophages. (A) Fold variations of NFATc1 and Tlr4 mRNAs measured by quantitative real time PCR in BMMs transfected with the NFATc1a siRNA, or with a negative control (Control) siRNA and compared to non-transfected BMMs. (B and C) Fold variation of Tlr4 mRNA expression measured by quantitative real-time PCR in WT BMMs incubated with or without 1 µM 11R-VIVIT (B and C) or without or with CsA for 48 h, then with or without 2 µM ionomycin for additional 2 h (C). Values are presented as mean ± SE from 3 independent experiments. *, p<0.05 (Two-tailed, unpaired Student's t test). (TIF) [file ppat.1003152.s006.tif]
