# Supplementary material for: Cyclosporine A Impairs Nucleotide Binding Oligomerization Domain (Nod1)-Mediated Innate Antibacterial Renal Defenses in Mice and Human Transplant Recipients
Source: PLoS Pathog. 2013 Jan 31;9(1):e1003152. doi: 10.1371/journal.ppat.1003152 (PMC3561241; doi:10.1371/journal.ppat.1003152)
Supplement: Table S1 — Demographic characteristics and biological parameters of the renal transplant recipients analyzed. (DOC) [file ppat.1003152.s007.doc]

**Table S1.**

Demographic characteristics and biological parameters of the renal transplant recipients analyzed

| Number of patients | 25 |
| --- | --- |
| Rank of transplantation | 1.02 ± 0.02 |
| Post-transplant follow up (months) | 22.2 ± 2.1 |
| Recipient age (years) | 53.2 ± 2.4 |
| Ethnic group (Caucasian/African) | 11 / 14 |
| Male/ Female recipients | 19 / 6 |
| Initial end-stage renal disease |  |
| Glomerulonephritis | 10 |
| Nephroangiosclerosis and others | 15 |
| Donor age (years) | 55.0 ± 1.7 |
| Deceased donor | 30 (100%) |
| Male/ Female donor | 14 / 12 |
| Cold-ischemia time (h) | 15.7 ± 0.6 |
| Biological parameters |  |
| GFR (ml/min/1.73m^2^) | 55.1 ± 1.8 |
| CRP (mg/l) | 2.6 ± 0.9 |
| Blood cell count (number/μl) |  |
| Leukocytes | 6370 ± 274 |
| Lymphocytes | 1520 ± 127 |
| Neutrophils | 4039 ± 287 |

GFR, Glomerular filtration rate; CRP, C reactive protein. When indicated values are expressed as mean ± SE.
