# Supplementary material for: Cyclosporine A Impairs Nucleotide Binding Oligomerization Domain (Nod1)-Mediated Innate Antibacterial Renal Defenses in Mice and Human Transplant Recipients
Source: PLoS Pathog. 2013 Jan 31;9(1):e1003152. doi: 10.1371/journal.ppat.1003152 (PMC3561241; doi:10.1371/journal.ppat.1003152)
Supplement: Table S2 — Mouse and human primers and Taqman probes used for quantitative real-time PCR and reverse transcription PCR. (DOC) [file ppat.1003152.s008.doc]

**Table S2.**

Mouse and human primers and Taqman probes used for quantitative real-time PCR and reverse transcription PCR

| Real-time PCR | | | |
| --- | --- | --- | --- |
| Mouse gene | | Primer sequence | Taqman probe* |
| *Tlr2* | (NM_011905.3) | nt 1661-1681, nt 1730-1712 | nt 1683-1710 |
| *Tlr4* | (BC029856) | nt 2834-2854, nt 2906-2887 | nt 2856-2881 |
| *Tlr5* | (AF186107.1) | nt 1010-1035, nt 1087-1067 | nt 1037-1062 |
| *Tlr9* | (AF314224) | nt 2679-2699, nt 2751-2730 | nt 2701-2728 |
| *Nod1* | (BC042670) | nt 2611-2630, nt 2701-2683 | nt 2644-2676 |
| *Nod2* | (AY160220) | nt 2688-2707 nt 2756-2740 | nt 2710-2733 |
| *β-actin* | (NM_007393.3) | nt 693-712, nt 830-810 | nt 763-785 |

| Human gene | | Primer sequence | Taqman probe* |
| --- | --- | --- | --- |
| *TLR2* | (NM_003264.3) | nt 2339-2360, nt 2421-2400 | nt 2364-2392 |
| *TLR4* | (NM_138554.3) | nt 524-544, nt 601-580 | nt 548-578 |
| *NOD1* | (NM_006092.2) | nt 2875-2896, nt 2972-2948 | nt 2909-2939 |
| *NOD2* | (NM_022162.1) | nt 2558-2578, nt 2679-2658 | nt 2595-2618 |
| *GAPDH* | (NM_002046.3) | nt 108-126, nt 333-314 | nt 285-304 |

*FAMTAMRA probes

| Reverse transcription PCR | | |
| --- | --- | --- |
| Mouse gene Primer sequence | | |
| *NFATc1* | (NM_016791) | nt 1593-1611, nt 2009-1989 |
| *Tlr4* | (NM_021297) | nt 1787-1808, nt 2097-2076 |
| *Nod1* | (NM_172729) | nt 1389-1412, nt 1913-1892 |
| *Nod2* | (NM_145857) | nt 947-968, nt 1453-1432 |
| *GAPDH* | (AK144690) | nt 307-327, nt 663-643 |
